# ﻿From 153-year-old records to contemporary discoveries: land snail (Mollusca, Gastropoda) diversity in Penang Hill, Malaysia

**DOI:** 10.3897/zookeys.1230.136906

**Published:** 2025-03-05

**Authors:** Soo-Mun Goh, Dulipat Jasrul, Mei-Yi Lee, Jaap J. Vermeulen, Thor-Seng Liew

**Affiliations:** 1 Institute for Tropical Biology and Conservation, Universiti Malaysia Sabah, Kota Kinabalu, Malaysia Universiti Malaysia Sabah Kota Kinabalu Malaysia; 2 School of Biological Science, Universiti Sains Malaysia, Penang, Malaysia Universiti Sains Malaysia Penang Malaysia; 3 jk.artandscience, Lauwerbes 8, 2318 AT, Leiden, Netherlands jk.artandscience Leiden Netherlands

**Keywords:** Bukit Bendera, Gastropoda, Island, Malay Peninsula, Pulau Pinang, semi-slugs, slugs

## Abstract

This study highlights the diversity of land snails in Penang Hill, a non-limestone hill in Peninsular Malaysia. A systematic survey of land snails in Penang Hill was conducted, inventoried, and compared with those specimens collected by Stoliczka (1872, 1873) in 1869. Based on the 33 sampling plots established in this study, the differences in species composition along the elevation gradient (75 m to 770 m a.s.l.) and between three different habitats on Penang Hill were examined: disturbed forests with anthropogenic activities, undisturbed forests, and orchards with various crops. A total of 54 species were recorded from the sampling plots and random observations, of which only 20 overlapped with Stoliczka’s list; 34 were new records for Penang Hill, and 12 previously recorded species were not found in this study. Most of the new records were micro-snails smaller than 5 mm. Species richness was highest in disturbed forests and showed no correlation with elevation. There was no clear grouping of plots by species composition across habitat types, except for those in orchards. Indicator species analysis revealed strong associations between a few land snail species and disturbed forests and orchards. The number of species in Penang Hill increased from 32 to 66, with species richness comparable to the high numbers usually found in limestone habitats and higher mountains in Malaysia.

## ﻿Introduction

The history of land snail research in Peninsular Malaysia began during the establishment of the Straits Settlements in 1826, which at that time consisted of Penang Island, Singapore, and Malacca. The first land snail species described from the Malay Peninsula islands was *Quantulastriata* (Gray, 1834) from Singapore, while *Dyakiamackensiana* (Souleyet, 1841) was described from the Malay Peninsula mainland in Malacca (Gray 1834; Souleyet 1841). Since then, more land snail species have been described from these areas: another 15 species from the Malay Peninsula mainland (Souleyet 1841, 1842; Sowerby 1842; Pfeiffer 1855a, 1855b, 1857); six species from Penang Island (Benson 1851a, 1851b, 1852a, 1861; Souleyet 1852; Pfeiffer 1856); and another six species from Singapore (Sowerby 1843; Adams and Adams 1851; Pfeiffer 1851, 1853, 1855c; Benson 1852b). Most of the species described in the above literature were from non-systematic sampling. The first detailed land snail inventory study was conducted by Stoliczka, who explored the northwestern part of Penang Island, mainly Penang Hill, for 16 days in 1869, and recorded a total of 32 species. He also described 19 new species from Penang Hill, which was close to the total number of species described in the previous three decades in this region.

Despite the earlier works, all of which were from non-limestone areas, most later land snail inventory studies have largely focused on limestone hills, with little attention paid to non-limestone areas (de Morgan 1885a, 1885b; van Benthem Jutting 1950, 1952, 1954, 1960; Davison 1991; Clements et al. 2008; Liew et al. 2008; Foon et al. 2017; Phung et al. 2018; Foon and Marzuki 2023). For the studies conducted in non-limestone areas, most were based on opportunistic sampling that may not be sufficient to capture the true extent of land snail diversity, and comparative analysis could not be done (Chan 1998a, 1998b). Moreover, valuable information from these scattered inventory studies is often buried in the taxonomic literature (von Möllendorff 1886, 1891; Collinge 1902; Sykes 1903).

Land snail populations occur in low densities in non-limestone areas (Schilthuizen et al. 2003). This gives the impression that lowlands in non-limestone areas have very low land snail diversity (Liew et al. 2008). To date, most of the previous records of land snails in non-limestone areas are from former British colonial hill stations, namely Maxwell Hill (i.e., Bukit Larut), the Highlands of Telom Valley (i.e., Cameron Highlands), and Penang Hill, and from recent studies, namely Lojing Highlands and Temengor Forest Reserve (Stoliczka 1872, 1873; van Benthem Jutting 1949; Davison 1995; Liew 2010).

Stoliczka’s work remains a historical benchmark for the study of land snails in non-limestone areas. In the last 150 years, climate and habitat changes have likely affected land snail populations on Penang Hill, hence a contemporary study with systematic sampling is necessary. In particular, the presence of a small pocket of lower montane habitats at the top of the Penang Hills, due to the Massenerhebung effect (at ca 600–800 m a.s.l.), deserves attention, as such habitats that are normally found at higher elevations on the mainland have a smaller geographical extent on tropical islands. Furthermore, the habitat around Penang has changed significantly due to land use and the increase in tourism activities. To provide a comparison with the historical data collected by Stoliczka in the late 19^th^ century, we conducted a systematic survey of land snails on Penang Hill and compared the inventory with previously published lists by Stoliczka (1872, 1873). We then examined the land snail assemblages in three habitats on Penang Hill, namely disturbed forests with anthropogenic activities, undisturbed forests, and orchards with different crops. Finally, we investigated whether there are differences in land snail composition along the elevation gradient of Penang Hill.

## ﻿Materials and methods

### ﻿Study sites and sampling design

**Figure 1.**
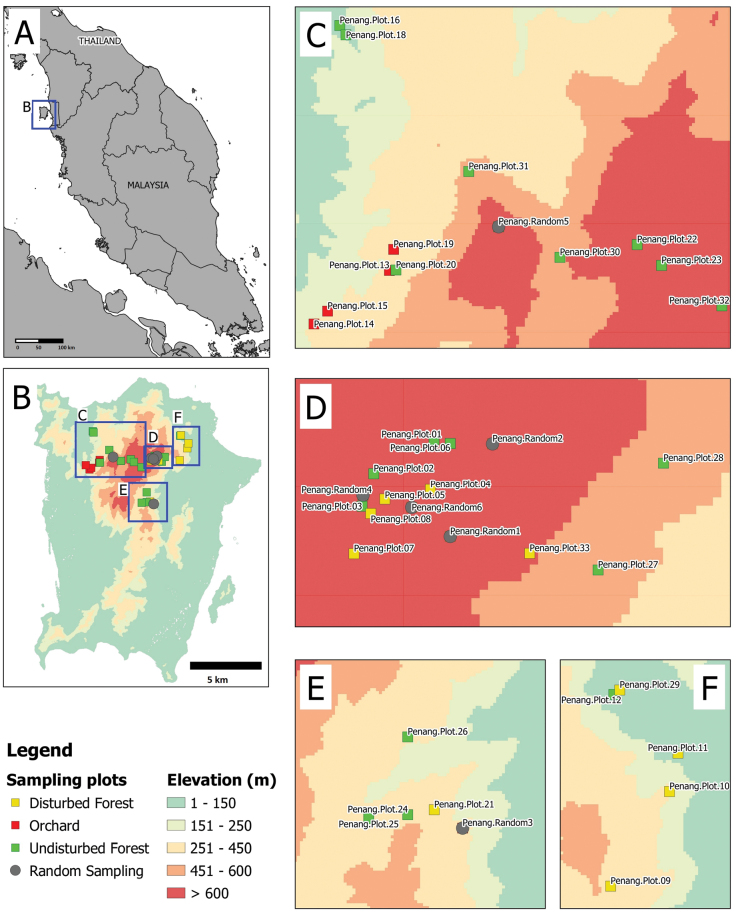
The locations sampling plots and locations on Penang Hill. The details of each of the 33 standard plots and the six random locations are given in Table 1**A** the location of Penang in Malaysia **B** an overview of sampling plots and locations at Penang Hill **C–F** the detailed distribution of the sampling plots and locations of the three habitat types at different elevations of this study.

A total of 33 standard plots were established for systematic sampling (Table 1, Fig. 1). Three habitat types with different degrees of habitat degradation: (1) forest with almost no anthropogenic activities (hereafter “Undisturbed Forest”); (2) moderately degraded habitat - disturbed forest with signs of anthropogenic activities such as man-made physical structures and altered vegetation structures (hereafter “Disturbed Forest”); and (3) highly degraded habitat – forest has been replaced by farmland and orchard (hereafter “Orchard”). The plots in disturbed and undisturbed forest can be divided into two altitudinal zones, lowland forest (< 600 m a.s.l.) and lower montane forest (> 600 m a.s.l.). Furthermore, any land snails found outside the standard sampling plots at six random locations were also collected for the species inventory list.

**Table 1.** Details of the 33 standard sampling plots and six random sampling locations, and the number of species and specimens recorded for each of the plots and sites.

Plot number	Location description	Plot	Elevation (m a.s.l.)	Habitat type	Latitude (dd), Longitude (dd)	Number of land snails species	Total number of specimens
Penang.Plot.01	Penang Hill. The Habitat. Research Trail.	Standard Sampling	720	Undisturbed Forest	5.42484, 100.26695	9	23
Penang.Plot.02	Penang Hill. The Habitat. Research Trail.	Standard Sampling	720	Undisturbed Forest	5.42371, 100.26494	2	3
Penang.Plot.03	Penang Hill. The Habitat. Research Trail.	Standard Sampling	750	Undisturbed Forest	5.42262, 100.26455	7	10
Penang.Plot.04	Penang Hill. The Habitat. Habitat nature trail.	Standard Sampling	770	Disturbed Forest	5.42318, 100.26683	10	55
Penang.Plot.05	Penang Hill. The Habitat. Habitat nature trail.	Standard Sampling	770	Disturbed Forest	5.42286, 100.26531	13	39
Penang.Plot.06	Penang Hill. The Habitat. Research Trail.	Standard Sampling	750	Undisturbed Forest	5.42471, 100.26749	10	41
Penang.Plot.07	Penang Hill. The Habitat. Habitat nature trail.	Standard Sampling	760	Disturbed Forest	5.42105, 100.26428	14	79
Penang.Plot.08	Penang Hill. The Habitat. Habitat nature trail.	Standard Sampling	760	Disturbed Forest	5.42238, 100.26484	9	28
Penang.Plot.09	Penang Hill. Moon Gate Station 5 trail.	Standard Sampling	410	Disturbed Forest	5.4216, 100.28575	1	1
Penang.Plot.10	Penang Hill. Moon Gate Station 5 trail.	Standard Sampling	250	Disturbed Forest	5.4305, 100.29133	4	7
Penang.Plot.11	Penang Hill. Moon Gate Station 5 trail.	Standard Sampling	110	Disturbed Forest	5.43411, 100.29213	9	34
Penang.Plot.12	Penang Hill. Botanical garden.	Standard Sampling	85	Undisturbed Forest	5.43967, 100.28605	7	14
Penang.Plot.13	Penang Hill. Teluk Bahang- Balik Pulau. Lam durian farm.	Standard Sampling	290	Orchard	5.41988, 100.22592	5	15
Penang.Plot.14	Penang Hill. Teluk Bahang- Balik Pulau. Tropical Fruit Farm.	Standard Sampling	260	Orchard	5.41484, 100.21877	4	26
Penang.Plot.15	Penang Hill. Teluk Bahang- Balik Pulau. Tropical Fruit Farm.	Standard Sampling	250	Orchard	5.41605, 100.22006	9	37
Penang.Plot.16	Penang Hill. Teluk Bahang. Taman Rimba Teluk Bahang. Trail. Simpang 6.	Standard Sampling	120	Undisturbed Forest	5.44297, 100.22122	10	22
Penang.Plot.17	Penang Hill. Teluk Bahang-Balik Pulau. Entrance of a durian farm, near Boulder Valley. Old rubber tree.	Standard Sampling	180	Orchard	5.418, 100.21598	9	19
Penang.Plot.18	Penang Hill. Teluk Bahang. Taman Rimba Teluk Bahang. Trail. Stesen 3.	Standard Sampling	150	Undisturbed Forest	5.44209, 100.2218	4	8
Penang.Plot.19	Penang Hill. Teluk Bahang- Balik Pulau. Lam durian farm.	Standard Sampling	280	Orchard	5.42186, 100.22632	5	17
Penang.Plot.20	Penang Hill. Teluk Bahang- Balik Pulau. Bukit Kerajaan ForestReserve next to Lam durian farm.	Standard Sampling	330	Undisturbed Forest	5.41992, 100.22654	4	4
Penang.Plot.21	Penang Hill. Air Itam. Forest near farm.	Standard Sampling	340	Disturbed Forest	5.39087, 100.26371	4	6
Penang.Plot.22	Penang Hill. Trail to Western hill. Junction to Teluk Bahang from Penang Hill.	Standard Sampling	760	Undisturbed Forest	5.42231, 100.2494	3	3
Penang.Plot.23	Penang Hill. Trail to Western hill. Km 3.6.	Standard Sampling	750	Undisturbed Forest	5.42035, 100.25171	6	16
Penang.Plot.24	Penang Hill. Air Itam. Forest near Dam.	Standard Sampling	380	Undisturbed Forest	5.3904, 100.2612	11	30
Penang.Plot.25	Penang Hill. Air Itam. Forest near Dam.	Standard Sampling	290	Undisturbed Forest	5.39003, 100.2575	8	32
Penang.Plot.26	Penang Hill. Air Itam. Forest near Dam.	Standard Sampling	290	Undisturbed Forest	5.39772, 100.2612	7	19
Penang.Plot.27	Penang Hill. Trail from Viaduct to Claremont, after shelter.	Standard Sampling	500	Undisturbed Forest	5.42051, 100.27242	8	12
Penang.Plot.28	Penang Hill. Trail - Moniot Road East.	Standard Sampling	530	Undisturbed Forest	5.42406, 100.2746	11	29
Penang.Plot.29	Penang Hill. Botanical garden.	Standard Sampling	75	Disturbed Forest	5.44009, 100.28661	15	38
Penang.Plot.30	Penang Hill. Western hill to Teluk Bahang Trail Stetion 4	Standard Sampling	570	Undisturbed Forest	5.42111, 100.24208	1	1
Penang.Plot.31	Penang Hill. Western hill to Teluk Bahang Trail Stetion 7	Standard Sampling	490	Undisturbed Forest	5.42919, 100.23344	1	1
Penang.Plot.32	Penang Hill. Moniot Trail	Standard Sampling	695	Undisturbed Forest	5.41655, 100.25743	6	10
Penang.Plot.33	Penang Hill. By Path H	Standard Sampling	610	Disturbed Forest	5.42106, 100.27014	16	90
Penang.Random1	Penang Hill. Path B.	Random Sampling	730	Disturbed Forest	5.42162, 100.26749	8	15
Penang.Random2	Penang Hill. Plaza.	Random Sampling	740	Disturbed Forest	5.4247, 100.2689	1	1
Penang.Random3	Penang Hill. Air Itam Farm. Batu Panay road.	Random Sampling	270	Orchard	5.38914, 100.2664	1	5
Penang.Random4	Penang Hill. The Habitat. Research Trail.	Random Sampling	740	Undisturbed Forest	5.42299, 100.26458	1	1
Penang.Random5	Penang Hill. Bukit Laksamana	Random Sampling	710	Undisturbed Forest	5.42399, 100.23627	1	1
Penang.Random6	Penang Hill. Along Jalan Tunku Yahya Petra	Random Sampling	730	Disturbed Forest	5.422586082, 100.266207	3	11

Research permits were obtained from Penang Forestry Department – JPNPP/600-9/1 Jld.3(47), Forestry Department Peninsular Malaysia – JH/100 Jld.34(8), Penang Botanic Gardens – JTB/pp/02/03/032 Jld.5(7), and Penang Water Supply Corporation – PBAPP/CAD/SC/#EXT/1/05/14(6).

### ﻿Land snail sampling and identification

Each standard plot, an area of 20 m × 20 m, was searched by two persons for one hour, and a total of 5 litres of soil and leaf litter was collected. The sampling was conducted on the following dates: 4–7 Sept 2022, 21–26 Oct 2022, 2–6 Feb 2023, and on 20 Jun 2023. Afterwards, the snails were cleaned and preserved, and the micro-snails (species smaller than 5 mm) were extracted from the soil and leaf litter samples (Foon et al. 2017). After that, the species were identified by comparing the material with illustrations and description in the literature. Where a species could not be identified with certainty, a working morphospecies name (for example, “sp.”, or “Penang sp. 1”) was used after the genus name. All authorities are provided in the checklist. All specimens were catalogued in the BORNEENSIS Mollusca collection database and were deposited in the BORNEENSIS collection of Institute for Tropical Biology and Conservation, Universiti Malaysia Sabah: BOR/MOL 15081 – BOR/MOL 15164, BOR/MOL 15195 – BOR/MOL 15201, BOR/MOL 15217 – BOR/MOL 15250, BOR/MOL 15268 – BOR/MOL 15305, BOR/MOL 15412 – BOR/MOL 15486, BOR/MOL 15539 – BOR/MOL 15566, BOR/MOL 15726 – BOR/MOL 15773.

### ﻿Data analysis

For the compilation of the species list, the data from the standard sampling plots and random sites were combined with the records of Stoliczka (1872, 1873). We also checked all the snail photos uploaded by citizen naturalists at iNaturalist. We filtered the records for the geographical extent of Penang Hill, and for Mollusca (https://www.inaturalist.org/observations?captive=false&nelat=5.474271882208513&nelng=100.29576600820225&photos&place_id=any&subview=table&swlat=5.364899735652405&swlng=100.17491639882725&verifiable=any&iconic_taxa=Mollusca). After compiling the list, we checked the status of each species, alien or native. We also gathered distribution data for each species to assess the likelihood that it is endemic to Penang Hill.

For the other biodiversity analysis, only the data from the standard sampling plots were used. The number of species (species richness) and the number of specimens of each species (abundance) were tabulated for each standard sampling plot. The sampling completeness and the estimate of total species richness based on the data from the standard sampling plots were determined using the iNEXT package in R.

For comparison of species richness and fauna composition among plots of different habitat types, we test the hypothesis that there are no differences in species richness among the three habitat types and the species richness is not correlated with elevation. As the normality of species richness and homogeneity of variance between habitat types did not violate the test assumptions, we tested the hypothesis by using a one-way ANCOVA. The response variable is species richness of the plots, with the habitat types as different groups and elevation as a cofounding factor. To investigate whether fauna composition is determined by habitat types and/or elevation, we conducted two analyses to identify underlying patterns of land snail composition. First, we conducted a cluster analysis based on absence and presence data (Jaccard index) of the 50 species in the 33 plots. Then we performed non-metric multidimensional scaling (NMDS) based on the absence/presence data (Jaccard’s distance). NMDS analysis is a dimensionality reduction technique used to visualise and explore similarities, while cluster analysis is used to identify natural groupings based on similarity of land snail composition. Finally, we conducted an indicator species analysis (package “indicspecies”) to identify species significantly associated with each of the three habitat types (Cáceres and Legendre 2009). All analyses were done with R.

## ﻿Results

### ﻿Sampling completeness and inventory of land snails

Our samples yielded 54 land snail species, with a total of 803 specimens. Altogether, 50 species were found in the 33 standard sampling plots, while the remaining four species were found in random and opportunistic sampling locations. Of these 54 land snail species, 34 represent new records compared to the lists of Stoliczka (1872, 1873). We did not recover 12 species recorded previously by Stoliczka, although one of these species (*Meghimatiumpictum*) has recently been recorded a few times by citizen naturalists (https://www.inaturalist.org/observations/102000427). Since the work of Stoliszca, the total number of recorded land snail species from Penang has increased from 32 species to 66 species (Table 2). Among the 34 species additional to Stoliczka’s list, 19 species are micro-snails (species smaller than 5 mm). They belong to the genera *Kaliella* (6 species), *Microcystina* (5 species), *Philalanka* (2 species), *Diplommatina* (2 species), *Charopa* (2 species), and one species each of *Ditropopsis* and *Paralaoma*. The remaining seven species are commonly found garden snails and slugs, namely, *Allopeasclavulinum*, *Laevicaulisalte*, *Lissachatinafulica*, *Macrochlamysindica*, *Macrochlamystersa*, *Parmarionmartensi*, and *Subulinaoctona*. These species are identified as alien species.

**Table 2.** Comparison of the species list between the previous study by Stoliczka (1872, 1873) and this study.

Family	Species	Stoliczka (1872, 1873)	This study
Alycaeidae	*Alycaeusgibbosulus* Stoliczka, 1872	1	0
Cyclophoridae	*Cyclophorusmalayanus* (Benson, 1852)	1	1
Cyclophoridae	*Cyclophorusperdixborneensis* (Metcalfe, 1854)	1	1
Cyclophoridae	*Cyclotussolutus* (Stoliczka, 1872)	1	0
Cyclophoridae	*Ditropopsis* sp.	0	1
Cyclophoridae	*Lagocheilus* Penang sp. 2	0	1
Cyclophoridae	*Lagocheilustrochoides* (Stoliczka, 1872)	1	1
Cyclophoridae	*Lagocheilusstriolatus* Stoliczka, 1872	1	1
Cyclophoridae	*Opisthoporuspenangensis* (Stoliczka, 1872)	1	1
Diplommatinidae	*Diplommatina* Penang sp. 1	0	1
Diplommatinidae	*Diplommatinacrosseana* Godwin-Austen & Nevill, 1879	0	1
Pupinidae	*Coptocheilussectilabris* (A. Gould, 1844)	1	1
Pupinidae	*Pupinaaureola* Stoliczka, 1872	1	1
Pupinidae	*Rhaphauluslorraini* L. Pfeiffer, 1856	0	1
Achatinidae	*Allopeasclavulinum* (Potiez & Michaud, 1838)	0	1
Achatinidae	*Allopeasgracile* (T. Hutton, 1834)	1	1
Achatinidae	*Lissachatinafulica* (Bowdich, 1822)	0	1
Achatinidae	*Paropeastchehelense* (de Morgan, 1885)	0	1
Achatinidae	*Subulinaoctona* (Bruguière, 1792)	0	1
Philomycidae	*Meghimatiumpictum* (Stoliczka, 1873)	1	0
Clausiliidae	*Oospirapenangensis* (Stoliczka, 1873)	1	1
Clausiliidae	*Phaedusafilicostata* (Stoliczka, 1873)	1	0
Ariophantidae	*Hemiplectacymatium* (L. Pfeiffer, 1856)	1	1
Ariophantidae	*Microcystina* Penang sp. 1	0	1
Ariophantidae	*Microcystina* Penang sp. 2	0	1
Ariophantidae	*Microcystina* Penang sp. 4	0	1
Ariophantidae	*Microcystina* Penang sp. 5	0	1
Ariophantidae	*Microcystina* Penang sp. 6	0	1
Ariophantidae	*Parmarionmartensi* Simroth, 1893	0	1
Ariophantidae	*Tanychlamysindica* Godwin-Austen, 1883	0	1
Ariophantidae	*Tanychlamys* Penang sp. 1	0	1
Ariophantidae	*Tanychlamys* Penang sp. 2	0	1
Ariophantidae	*Tanychlamysstephoides* Stoliczka, 1873	1	1
Ariophantidae	*Tanychlamystersa* (Issel, 1874)	0	1
Helicarionidae	*Helicarionpermolle* Stoliczka, 1873	1	1
Camaenidae	*Amphidromusatricallosus* (A. Gould, 1843)	1	0
Camaenidae	*Amphidromusperversus* (Linnaeus, 1758)	1	0
Camaenidae	*Bradybaenasimilaris* (A. Férussac, 1821)	1	1
Camaenidae	*Trichochloritispenangensis* (Stoliczka, 1873)	1	1
Charopidae	*Charopaperlata* van Benthem Jutting, 1959	0	1
Charopidae	*Charopa* Penang sp. 2	0	1
Charopidae	*Philalankacarinifera* (Stoliczka, 1873)	1	1
Charopidae	*Philalanka* Penang sp. 2	0	1
Charopidae	*Philalankakusana* (Aldrich, 1889)	0	1
Punctidae	*Paralaoma* sp.	0	1
Gastrocoptidae	*Gastrocoptapalmira* (Stoliczka, 1873)	1	0
Valloniidae	*Pupisomaorcella* (Stoliczka, 1873)	1	0
Streptaxidae	*Gulellabicolor* (T. Hutton, 1834)	1	0
Chronidae	*Kaliellabarrakporensis* (Pfeiffer, 1852)	0	1
Chronidae	*Kaliella* Penang sp. 1	0	1
Chronidae	*Kaliella* Penang sp. 2	0	1
Chronidae	*Kaliella* Penang sp. 4	0	1
Chronidae	*Kaliella* Penang sp. 5	0	1
Chronidae	*Kaliellascandens* (Cox, 1872)	0	1
Microcystidae	*Microcystispalmicola* Stoliczka, 1873	1	0
Chronidae	*Vitrinopsis* sp.	0	1
Chronidae	*Vitrinopsisnucleata* (Stoliczka, 1873)	1	1
Dyakiidae	*Quantulastriata* (J. E. Gray, 1834)	0	1
Dyakiidae	*Pseudoplectabijuga* (Stoliczka, 1873)	1	1
Trochomorphidae	*Videnacastra* (Benson, 1852)	1	1
Trochomorphidae	*Videnacantoriana* (W. H. Benson, 1861)	1	0
Trochomorphidae	*Videnatimorensis* (E. von Martens, 1867)	1	1
Rathouisiidae	*Atopostourannensis* (Souleyet, 1852)	1	1
Rathouisiidae	*Atopospunctata* Collinge, 1902	0	1
Veronicellidae	*Laevicaulisalte* (A. Férussac, 1822)	0	1
Veronicellidae	*Semperulabirmanica* (Theobald, 1864)	1	0

The land snail diversity across the sampling plots shows several patterns. Among the 33 plots, nine contained between 10 and 16 species, while 14 plots had 5–9 species, seven plots had 2–4 species, and three plots had only one species. Four species emerged as the most common, being found in more than half of the sampling plots: *Pseudoplectabijuga* (recorded in 22 plots), *Cyclophorusmalayanus* (found in 17 plots), *Macrochlamys* Penang sp. 1 (present in 17 plots), and *Microcystina* Penang sp. 1 (observed in 17 plots). The latter two were not recorded by Stoliczka.

**Figure 2.**
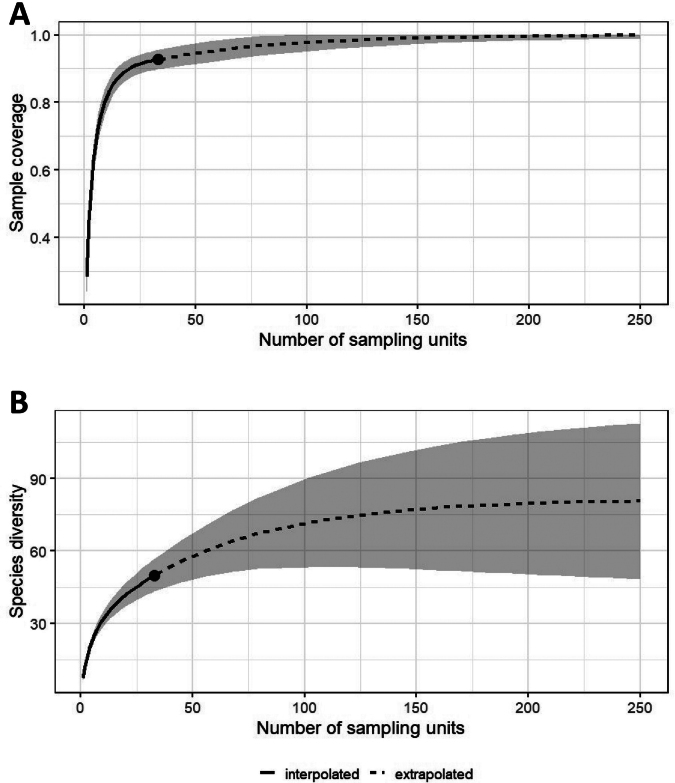
The sample completeness curve based on the 33 standard plots (black dot, i.e., sampling units). Plots of sample coverage for interpolated (solid line) and extrapolated sampling plots (dashed line). The 95% confidence intervals (shaded areas) were obtained by a bootstrap method based on 1000 replications. Each of the two curves was extrapolated to up to 250 plots **A** sampling coverage **B** species diversity (richness) estimated based on the extrapolated samples.

In contrast, 28 species displayed limited distributions, being found in not more than three sampling plots (10% of the 33 plots). Among these species, 18 were singleton species; they were found in a single plot only. Additionally, five species were found in only two plots, and five species were recorded in just three plots. Altogether ten of the 34 species not recorded by Stolizka were singleton species. The sampling coverage for the 33 plots was determined to be 92.3%, with 50 observed species (Fig. 2). Based on species richness estimates with rarefied and extrapolated samples, the further extrapolation resulted in the expectation of 94 species.

### ﻿Species richness and composition among plots of different habitat types

Disturbed forest seems to have the highest species richness per plot: undisturbed forest (mean ± s.d.: 6.4 ± 3.3 species), disturbed forest (9.5 ± 5.1 species), and orchards (6.4 ± 2.4 species), but it is not statistically significantly different (F = 2.206, df = 2.29, p = 0.128). Elevation did not have a confounding effect (F = 0.079, df = 1.29, p = 0.780).

**Figure 3.**
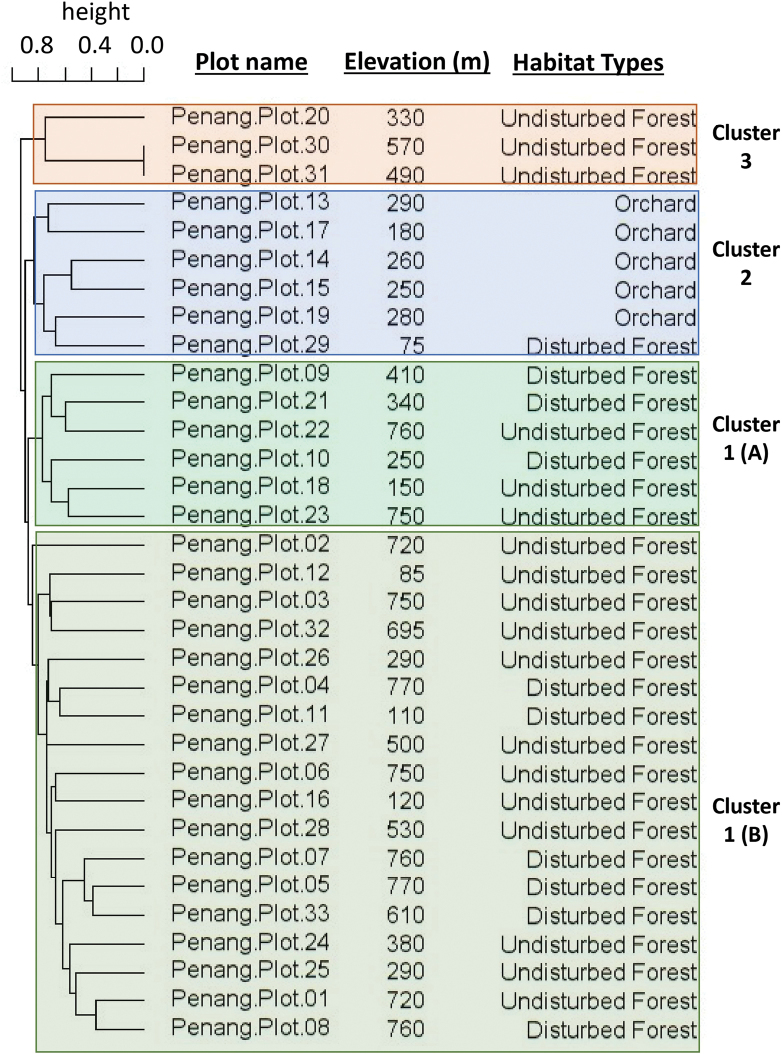
Cluster analysis based on Jaccard dissimilarity of absence and presence data of land snail species composition in 33 standard plots in Penang Hill, Penang Island, Peninsular Malaysia. Three main clusters were identified: Cluster 1, comprising two sub-clusters in which almost all plots in undisturbed and disturbed forests were grouped; Cluster 2, comprising all plots in orchards and one plot in a disturbed forest patch in the botanical garden; and Cluster 3, comprising three undisturbed plots.

Cluster analysis based on the absence/presence of 50 species in the 33 plots did not show a general pattern of clustering of plots with respect to elevation (Fig. 3). The plots in the different types of orchards showed some similarity in terms of species composition, and plot 29 in the small forest patch in the botanical garden was clustered with other plots in orchards (cluster 2 in Fig. 3). There is also no clear indication that the plots in disturbed and undisturbed forests differ in terms of species composition (cluster 1 and cluster 3 in Fig. 3).

**Figure 4.**
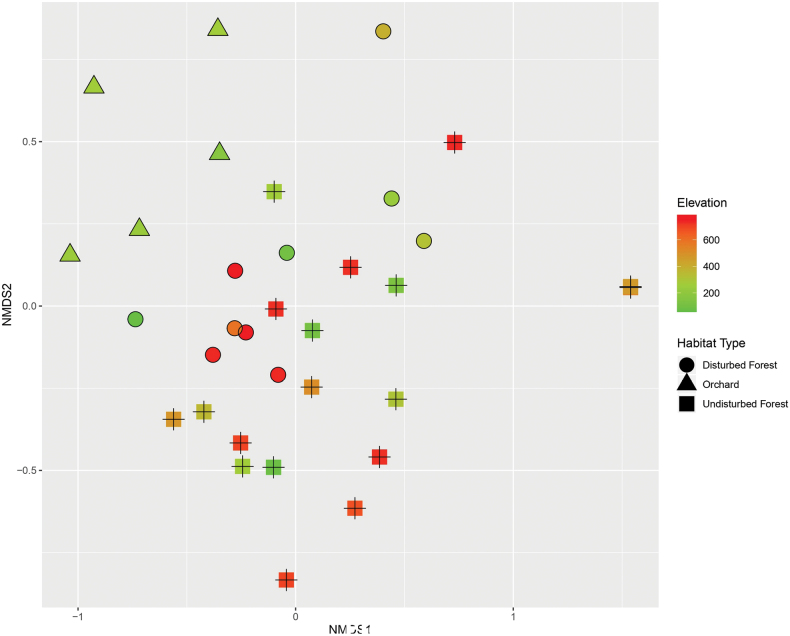
Non-metric multidimensional scaling (NMDS) ordination of land snail communities between the 33 standard plots based on Jaccard’s distance (stress value 0.19). Different symbols represent different habitat types. The colour of the symbols indicates the elevation of the plots.

To further investigate species composition between plots, we used NMDS to visualise and reduce the dimensionality of Jaccard’s distance matrices based on absence/presence data (Fig. 4). The stress value of 0.17 indicates that the NMDS plot is a fair representation of the data among plots. However, similar to the cluster analysis, there are no clear patterns for species composition in the plots of different habitat types and of different elevations, with the exception of plots in orchards.

The indicator species analysis revealed strong associations between certain land snail species and specific habitat types. *Helicarionpermolle* (value = 0.52, p = 0.024) and *Lissachatinafulica* (value = 0.618, p = 0.008) were found to be closely linked to disturbed forest areas, signifying their preference for such habitats. On the other hand, *Allopeasgracile* (value = 0.731, p = 0.002), *Kaliellascandens* (value = 0.567, p = 0.016), and *Charopa* Penang sp. 2 (value = 0.567, p = 0.014) exhibited strong affiliations with orchards, indicating their specific affinity for these modified landscapes.

### ﻿Checklist

#### ﻿Class Gastropoda Cuvier, 1795,

**Subclass Caenogastropoda Cox, 1960**

**Superfamily Cyclophoroidea J. E. Gray, 1847**

**Family Alycaeidae W. T. Blanford, 1864**

**Genus *Alycaeus* J. E. Gray, 1850**

##### ﻿ Alycaeus gibbosulus

Taxon classificationAnimaliaGastropodaAlycaeidae

Stoliczka, 1872

51C9047E-640E-5D53-B482-BCC921C62F21

###### Remark.

Old record from the base of Penang Hill by Stoliczka (1872).

#### ﻿Family Cyclophoridae J. E. Gray, 1847

**Genus *Cyclophorus* Montfort, 1810**

##### ﻿ Cyclophorus malayanus

Taxon classificationAnimaliaGastropodaCyclophoridae

(W. H. Benson, 1852)

8C872F22-5556-5ADE-BEA0-749CC3407493

Figs 5A , 19A

###### Material examined.

Malaysia • Penang Hill, The Habitat, Research trail, Plot.01; 5.4248°N, 100.2669°E; 720 m a.s.l.; 5 Sept. 2022; T.S. Liew, J. Dulipat, S.M. Goh leg.; BOR/MOL 15163 • Penang Hill, The Habitat, Habitat nature trail, Plot.04; 5.4231°N, 100.2668°E; 770 m a.s.l.; 5 Sept. 2022; T.S. Liew, J. Dulipat, S.M. Goh leg.; BOR/MOL 15119 • Penang Hill, The Habitat, Habitat nature trail, Plot.05; 5.4228°N, 100.2653°E; 770 m a.s.l.; 5 Sept. 2022; T.S. Liew, J. Dulipat, S.M. Goh leg.; BOR/MOL 15126 • Penang Hill, The Habitat, Research trail, Plot.06; 5.4247°N, 100.2674°E; 750 m a.s.l.; 5 Sept. 2022; T.S. Liew, J. Dulipat, S.M. Goh, M.Y. Lee, M.Y. Wua leg.; BOR/MOL 15140 • Penang Hill, The Habitat, Habitat nature trail, Plot.08; 5.4223°N, 100.2648°E; 760 m a.s.l.; 6 Sept. 2022; J. Dulipat, S.M. Goh, M.Y. Lee leg.; BOR/MOL 15099 • Penang Hill, Moon Gate Station 5 trail, Plot.11; 5.4341°N, 100.2921°E; 110 m a.s.l.; 7 Sept. 2022; T.S. Liew, J. Dulipat, S.M. Goh leg.; BOR/MOL 15100 • same data as for preceding; BOR/MOL 15101 • same data as for preceding; BOR/MOL 15152 • Penang Hill, Botanical garden, Plot.12; 5.4396°N, 100.2860°E; 85 m a.s.l.; 4 Sept. 2022; T.S. Liew, J. Dulipat, S.M. Goh leg.; BOR/MOL 15091 • Penang Hill, Teluk Bahang, Taman Rimba Teluk Bahang, Trail, Simpang 6, Plot.16; 5.4429°N, 100.2212°E; 120 m a.s.l.; 21 Oct. 2022; T.S. Liew, J. Dulipat, M.Y. Lee leg.; BOR/MOL 15221 • same data as for preceding; BOR/MOL 15273 • Penang Hill, Teluk Bahang-Balik Pulau, Entrance of a durian farm, near Boulder Valley, Old rubber tree, Plot.17; 5.418°N, 100.2159°E; 180 m a.s.l.; 22 Oct. 2022; T.S. Liew, J. Dulipat leg.; BOR/MOL 15222 • same data as for preceding; BOR/MOL 15278 • Penang Hill, Teluk Bahang- Balik Pulau, Bukit Kerajaan ForestReserve next to Lam durian farm, Plot.20; 5.4199°N, 100.2265°E; 330 m a.s.l.; 23 Oct. 2022; T.S. Liew, J. Dulipat, M.Y. Lee leg.; BOR/MOL 15281 • Penang Hill, Air Itam, Forest near Dam, Plot.24; 5.3904°N, 100.2612°E; 380 m a.s.l.; 24 Oct. 2022; T.S. Liew, J. Dulipat leg.; BOR/MOL 15219 • same data as for preceding; BOR/MOL 15232 • same data as for preceding; BOR/MOL 15289 • Penang Hill, Air Itam, Forest near Dam, Plot.25; 5.3900°N, 100.2575°E; 290 m a.s.l.; 25 Oct. 2022; T.S. Liew, J. Dulipat leg.; BOR/MOL 15295 • Penang Hill, Air Itam, Forest near Dam, Plot.26; 5.3977°N, 100.2612°E; 290 m a.s.l.; 25 Oct. 2022; T.S. Liew, J. Dulipat leg.; BOR/MOL 15240 • same data as for preceding; BOR/MOL 15296 • Penang Hill, Trail - Moniot Road East, Plot.28; 5.4240°N, 100.2746°E; 530 m a.s.l.; 26 Oct. 2022; T.S. Liew, J. Dulipat leg.; BOR/MOL 15298 • Penang Hill, Botanical garden, Plot.29; 5.4400°N, 100.2866°E; 75 m a.s.l.; 2 Feb. 2023; T.S. Liew, M.Y. Lee, A. Yusni leg.; BOR/MOL 15729 • same data as for preceding; BOR/MOL 15766 • Penang Hill, Moniot trail, Plot.32; 5.4165°N, 100.2574°E; 695 m a.s.l.; 4 Feb. 2023; T.S. Liew, A. Yusni leg.; BOR/MOL 15769 • Penang Hill, By Path H, Plot.33; 5.4210°N, 100.2701°E; 610 m a.s.l.; 4 Feb. 2023; T.S. Liew, A. Yusni leg.; BOR/MOL 15730 • Penang Hill, Path B, Random1. PathB; 5.4216°N, 100.2674°E; 730 m a.s.l.; 4 Sept. 2022; T.S. Liew, J. Dulipat leg.; BOR/MOL 15103 • same data as for preceding; BOR/MOL 15107 • same data as for preceding; BOR/MOL 15149.

**Figure 5.**
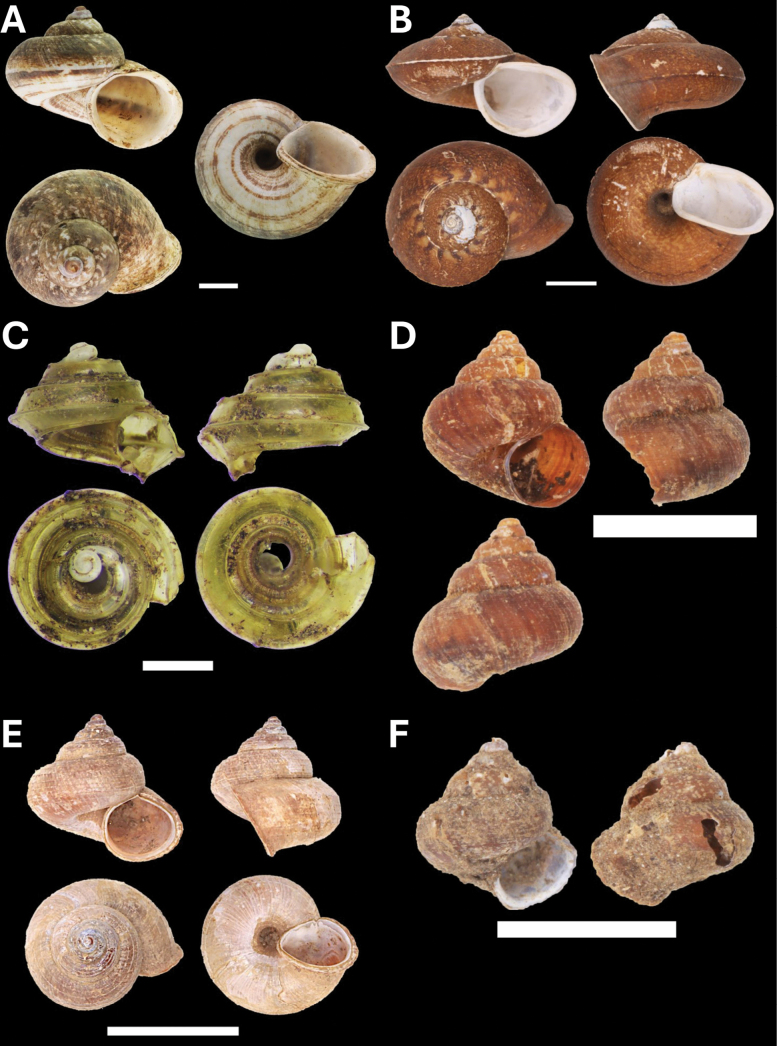
**A***Cyclophorusmalayanus* (W. H. Benson, 1852) BOR/MOL 15149 **B***Cyclophorusperdixborneensis* (Metcalfe, 1854) BOR/MOL 15726 **C***Ditropopsis* sp. BOR/MOL 15747 **D***Lagocheilus* Penang sp. 2 BOR/MOL 15429 **E***Lagocheilustrochoides* (Stoliczka, 1872) BOR/MOL 15739 **F***Lagocheilusstriolatus* Stoliczka, 1872 BOR/MOL 15200. Scale bars: 10 mm (**A, B**); 1 mm (**C**); 5 mm (**D–F**).

###### Remarks.

One of the most common large land snails at Penang Hill, with a shell width of up to 45 mm. It is also known from other localities in the northern part of Peninsular Malaysia. This species can be found in Perak as well (Foon et al. 2017).

##### ﻿ Cyclophorus perdix perdix

Taxon classificationAnimaliaGastropodaCyclophoridae

(Broderip & G. B. Sowerby I, 1830)

52C61D00-9CC8-53CA-8BD3-7B2D6CE7AE50

Figs 5B , 19B

###### Material examined.

Malaysia • Penang Hill, The Habitat, Research trail, Plot.03; 5.4226°N, 100.2645°E; 750 m a.s.l.; 5 Sept. 2022; T.S. Liew, J. Dulipat, S.M. Goh leg.; BOR/MOL 15157 • Penang Hill, The Habitat, Habitat nature trail, Plot.04; 5.4231°N, 100.2668°E; 770 m a.s.l.; 5 Sept. 2022; T.S. Liew, J. Dulipat, S.M. Goh leg.; BOR/MOL 15127 • same data as for preceding; BOR/MOL 15118 • Penang Hill, The Habitat, Habitat nature trail, Plot.05; 5.4228°N, 100.2653°E; 770 m a.s.l.; 5 Sept. 2022; T.S. Liew, J. Dulipat, S.M. Goh leg.; BOR/MOL 15135 • Penang Hill, The Habitat, Research trail, Plot.06; 5.4247°N, 100.2674°E; 750 m a.s.l.; 5 Sep . 2022; T.S. Liew, J. Dulipat, S.M. Goh, M.Y. Lee, M.Y. Wua leg.; BOR/MOL 15141 • Penang Hill, Teluk Bahang, Taman Rimba Teluk Bahang, Trail Stesen 3, Plot.18; 5.4420°N, 100.2218°E; 150 m a.s.l.; 22 Oct. 2022; T.S. Liew, J. Dulipat leg.; BOR/MOL 15282 • Penang Hill, Trail - Moniot Road East, Plot.28; 5.4240°N, 100.2746°E; 530 m a.s.l.; 26 Oct. 2022; T.S. Liew, J. Dulipat leg.; BOR/MOL 15299 • Penang Hill, Moniot trail, Plot.32; 5.4165°N, 100.2574°E; 695 m a.s.l.; 4 Feb. 2023; T.S. Liew, A. Yusni leg.; BOR/MOL 15728 • Penang Hill, By Path H, Plot.33; 5.4210°N, 100.2701°E; 610 m a.s.l.; 4 Feb. 2023; T.S. Liew, A. Yusni leg.; BOR/MOL 15726 • Penang Hill, Path B, Random1. PathB; 5.4216°N, 100.2674°E; 730 m a.s.l.; 4 Sept. 2022; T.S. Liew, J. Dulipat leg.; BOR/MOL 15102.

###### Remarks.

Less common than *Cyclophorusmalayanus* on Penang Hill, it was found to occur sympatrically with *Cyclophorusmalayanus* in this study. This species can also be found in Perak (Foon et al. 2017).

#### ﻿Genus *Cyclotus* Swainson, 1840

##### ﻿ Cyclotus solutus

Taxon classificationAnimaliaGastropodaCyclophoridae

(Stoliczka, 1872)

DA42C1C9-8190-55FC-A2B6-A09786B5F73F

###### Remarks.

This species is common in Perak (Foon et al. 2017) and was recorded by Stoliczka (1872) at the base of Penang Hill. However, it was not recorded in this study.

#### ﻿Genus *Ditropopsis* E. A. Smith, 1897

##### ﻿ Ditropopsis

Taxon classificationAnimaliaGastropodaCyclophoridae

sp.

1398AC9D-659B-574F-B1C6-5FD86CB7AD3E

Fig. 5C

###### Materials examined.

Malaysia • Penang Hill, Moniot trail, Plot.32; 5.4165°N, 100.2574°E; 695 m a.s.l.; 4 Feb. 2023; T.S. Liew, A. Yusni leg.; BOR/MOL 15747.

###### Remarks.

New record for Penang Hill. So far, only one *Ditropopsis* species has been recorded from Peninsular Malaysia (Maassen 2001), namely *Ditropopsiscavernae* (Sykes, 1903), which is distinctly different from the species found on Penang Hill. This genus was not recorded in previous systematic inventory studies in Peninsular Malaysia (Liew 2010; Foon et al. 2017; Foon and Marzuki 2023). The species from Penang Hill is similar to *Ditropopsisconstricta* Vermeulen, Liew & Schilthuizen, 2015 in terms of size, shape, and shell sculpture but differs by having a detached protoconch.

#### ﻿Genus *Lagocheilus* W. T. Blanford, 1864

##### ﻿ Lagocheilus

Taxon classificationAnimaliaGastropodaCyclophoridae

Penang sp. 2

2CF6B8FB-0AA1-5F90-BF56-62A0CA2672D0

Fig. 5D

###### Material examined.

Malaysia • Penang Hill, The Habitat, Habitat nature trail, Plot.04; 5.4231°N, 100.2668°E; 770 m a.s.l.; 5 Sept. 2022; T.S. Liew, J. Dulipat, S.M. Goh leg.; BOR/MOL 15430 • Penang Hill, The Habitat, Habitat nature trail, Plot.05; 5.4228°N, 100.2653°E; 770 m a.s.l.; 5 Sept. 2022; T.S. Liew, J. Dulipat, S.M. Goh leg.; BOR/MOL 15428 • Penang Hill, Moon Gate Station 5 trail, Plot.11; 5.4341°N, 100.2921°E; 110 m a.s.l.; 7 Sept. 2022; T.S. Liew, J. Dulipat, S.M. Goh leg.; BOR/MOL 15427 • Penang Hill, Teluk Bahang, Taman Rimba Teluk Bahang, Trail, Simpang 6, Plot.16; 5.4429°N, 100.2212°E; 120 m a.s.l.; 21 Oct. 2022; T.S. Liew, J. Dulipat, M.Y. Lee leg.; BOR/MOL 15541• Penang Hill, Teluk Bahang-Balik Pulau, Entrance of a durian farm, near Boulder Valley, Old rubber tree, Plot.17; 5.418°N, 100.2159°E; 180 m a.s.l.; 22 Oct. 2022; T.S. Liew, J. Dulipat leg.; BOR/MOL 15429.

###### Remarks.

This is a new record for Penang Hill. This species differs from *L.trochoides* by having more, less prominent spiral ribs, and from *L.striolatus* by having more spiral ribs. It also differs from *L.garelli* (Souleyet, 1852), which was previously recorded and described from Penang, by having more rapidly expanding whorl size.

##### ﻿ Lagocheilus trochoides

Taxon classificationAnimaliaGastropodaCyclophoridae

(Stoliczka, 1872)

6F1B86BB-7CB7-542F-BA1D-F08C795B5BEE

Figs 5E , 19C

###### Material examined.

Malaysia • Penang Hill, The Habitat, Research trail, Plot.01; 5.4248°N, 100.2669°E; 720 m a.s.l.; 5 Sept. 2022; T.S. Liew, J. Dulipat, S.M. Goh leg.; BOR/MOL 15081 • Penang Hill, The Habitat, Habitat nature trail, Plot.05; 5.4228°N, 100.2653°E; 770 m a.s.l.; 5 Sept. 2022; T.S. Liew, J. Dulipat, S.M. Goh leg.; BOR/MOL 15134 • Penang Hill, The Habitat, Research trail, Plot.06; 5.4247°N, 100.2674°E; 750 m a.s.l.; 5 Sept. 2022; T.S. Liew, J. Dulipat, S.M. Goh, M.Y. Lee, M.Y. Wua leg.; BOR/MOL 15084 • same data as for preceding; BOR/MOL 15085 • same data as for preceding; BOR/MOL 15139 • Penang Hill, The Habitat, Habitat nature trail, Plot.07; 5.4210°N, 100.2642°E; 760 m a.s.l.; 6 Sept. 2022; T.S. Liew, J. Dulipat, S.M. Goh, M.Y. Lee leg.; BOR/MOL 15143 • Penang Hill, The Habitat, Habitat nature trail, Plot.08; 5.4223°N, 100.2648°E; 760 m a.s.l.; 6 Sept. 2022; J. Dulipat, S.M. Goh, M.Y. Lee leg.; BOR/MOL 15156 • Penang Hill, Teluk Bahang, Taman Rimba Teluk Bahang, Trail, Simpang 6, Plot.16; 5.4429°N, 100.2212°E; 120 m a.s.l.; 21 Oct. 2022; T.S. Liew, J. Dulipat, M.Y. Lee leg.; BOR/MOL 15540 • Penang Hill, Teluk Bahang- Balik Pulau, Bukit Kerajaan ForestReserve next to Lam durian farm, Plot.20; 5.4199°N, 100.2265°E; 330 m a.s.l.; 23 Oct. 2022; T.S. Liew, J. Dulipat, M.Y. Lee leg.; BOR/MOL 15425 • Penang Hill, Air Itam, Forest near Dam, Plot.24; 5.3904°N, 100.2612°E; 380 m a.s.l.; 24 Oct. 2022; T.S. Liew, J. Dulipat leg.; BOR/MOL 15287 • Penang Hill, Trail from Viaduct to Claremont, after shelter, Plot.27; 5.4205°N, 100.2724°E; 500 m a.s.l.; 26 Oct. 2022; T.S. Liew, J. Dulipat leg.; BOR/MOL 15426 • Penang Hill, Botanical garden, Plot.29; 5.4400°N, 100.2866°E; 75 m a.s.l.; 2 Feb. 2023; T.S. Liew, M.Y. Lee, A. Yusni leg.; BOR/MOL 15738 • Penang Hill, By Path H, Plot.33; 5.4210°N, 100.2701°E; 610 m a.s.l.; 4 Feb. 2023; T.S. Liew, A. Yusni leg.; BOR/MOL 15737.

###### Remark.

This species is more common than *L.striolatus* at Penang Hill.

##### ﻿ Lagocheilus striolatus

Taxon classificationAnimaliaGastropodaCyclophoridae

Stoliczka, 1872

401E5B6D-FF73-5D3C-803A-740348E1586E

Fig. 5F

###### Material examined.

Malaysia • Penang Hill, The Habitat, Habitat nature trail, Plot.04; 5.4231°N, 100.2668°E; 770 m a.s.l.; 5 Sept. 2022; T.S. Liew, J. Dulipat, S.M. Goh leg.; BOR/MOL 15200.

###### Remark.

A rare species on Penang Hill.

#### ﻿Genus *Opisthoporus* W. H. Benson, 1851

##### ﻿ Opisthoporus penangensis

Taxon classificationAnimaliaGastropodaCyclophoridae

Stoliczka, 1872

5B4D3629-7881-51AC-9634-48341634BCDB

Fig. 6A

###### Material examined.

Malaysia • Penang Hill, Teluk Bahang, Taman Rimba Teluk Bahang, Trail, Simpang 6, Plot.16; 5.4429°N, 100.2212°E; 120 m a.s.l.; 21 Oct. 2022; T.S. Liew, J. Dulipat, M.Y. Lee leg.; BOR/MOL 15275.

**Figure 6.**
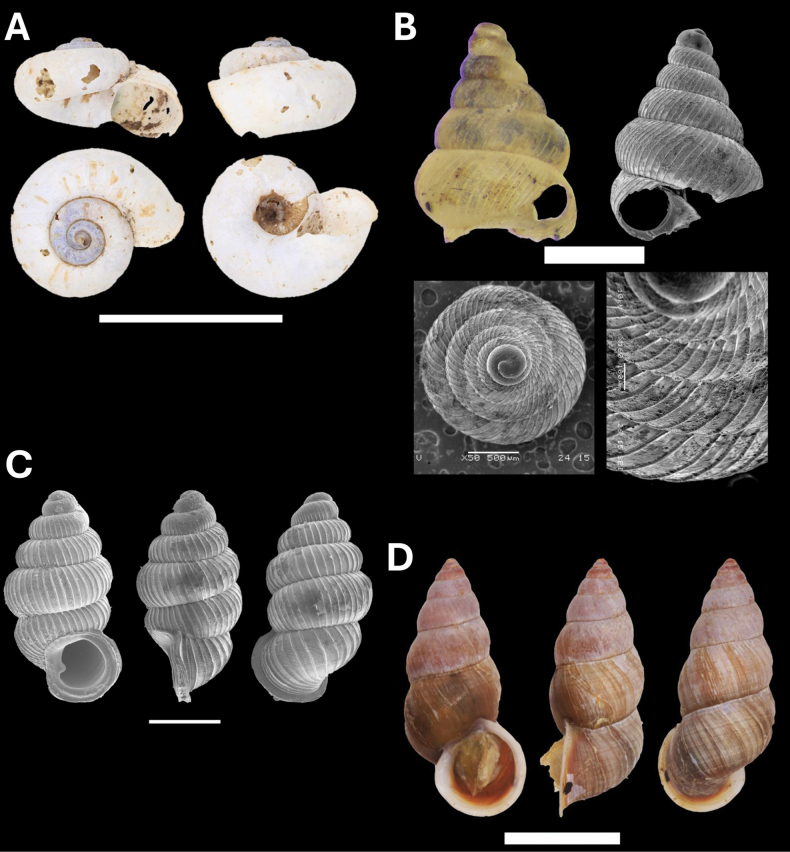
**A***Opisthoporuspenangensis* Stoliczka, 1872 BOR/MOL 15275 **B***Diplommatina* Penang sp. 1 BOR/MOL 15442 **C***Diplommatinacrosseana* Godwin-Austen & Nevill, 1879 BOR/MOL 15543 **D***Coptocheilussectilabris* (A. Gould, 1844) BOR/MOL 15270. Scale bars: 10 mm (**A, D**); 0.5 mm (**B, C**).

###### Remarks.

Originally described from base of Penang Hill (Stoliczka 1872) and recorded in only one plot of this study. This species has also been recorded in the northern part of Peninsular Malaysia and southern Thailand (Maassen 2001; Foon et al. 2017).

#### ﻿Family Diplommatinidae L. Pfeiffer, 1856

**Genus *Diplommatina* W. H. Benson, 1849**

##### ﻿ Diplommatina

Taxon classificationAnimaliaGastropodaDiplommatinidae

Penang sp. 1

E36BE821-E71C-5465-91E1-7C81610882B8

Fig. 6B

###### Material examined.

Malaysia • Penang Hill, Moon Gate Station 5 trail, Plot.10; 5.4305°N, 100.2913°E; 250 m a.s.l.; 7 Sept. 2022; T.S. Liew, J. Dulipat, S.M. Goh leg.; BOR/MOL 15442 • Penang Hill, Trail to Western hill, Km 3.6, Plot.23; 5.4203°N, 100.2517°E; 750 m a.s.l.; 24 Oct. 2022; T.S. Liew, J. Dulipat, M.Y. Lee leg.; BOR/MOL 15542.

###### Remarks.

This is a new record for Penang Hill. Based on the only shell fragments collected, it is likely *Diplommatinaventriculus*, which was originally described from Perak (von Möllendorff 1891).

##### ﻿ Diplommatina crosseana

Taxon classificationAnimaliaGastropodaDiplommatinidae

Godwin-Austen & Nevill, 1879

A4C1FB8D-692B-541C-AFB3-74C0846736D7

Fig. 6C

###### Material examined.

Malaysia • Penang Hill, Botanical garden, Plot.12; 5.4396°N, 100.2860°E; 85 m a.s.l.; 4 Sept. 2022; T.S. Liew, J. Dulipat, S.M. Goh leg.; BOR/MOL 15443 • Penang Hill, Teluk Bahang, Taman Rimba Teluk Bahang, Trail, Simpang 6, Plot.16; 5.4429°N, 100.2212°E; 120 m a.s.l.; 21 Oct. 2022; T.S. Liew, J. Dulipat, M.Y. Lee leg.; BOR/MOL 15543 • Penang Hill, Teluk Bahang, Taman Rimba Teluk Bahang, Trail Stesen 3, Plot.18; 5.4420°N, 100.2218°E; 150 m a.s.l.; 22 Oct. 2022; T.S. Liew, J. Dulipat leg.; BOR/MOL 15444 • Penang Hill, Air Itam, Forest near farm, Plot.21; 5.3908°N, 100.2637°E; 340 m a.s.l.; 23 Oct. 2022; T.S. Liew, J. Dulipat leg.; BOR/MOL 15544.

###### Remark.

New record for Penang Hill. Previously known only from Perak (Maassen 2001; Foon et al. 2017).

#### ﻿Family Pupinidae L. Pfeiffer, 1853

**Genus *Coptocheilus* A. Gould, 1862**

##### ﻿ Coptocheilus sectilabris

Taxon classificationAnimaliaGastropodaPupinidae

(A. Gould, 1844)

BFA47B62-A03E-59EB-BC5E-E76BBBB21EC8

Figs 6E , 19D

###### Material examined.

Malaysia • Penang Hill, The Habitat, Research trail, Random4.; 5.4229°N, 100.2645°E; 740 m a.s.l.; 27 Oct. 2022; M.Y. Lee leg.; BOR/MOL 15270.

###### Remarks.

So far, all the records were found at the top of Penang Hill, specifically around the Habitat, but it is very rare elsewhere on Penang Hill. It is a widespread species in Indochina and Peninsular Malaysia (Maassen 2001; Tumpeesuwan and Panha 2008; Foon et al. 2017).

#### ﻿Genus *Pupina* Vignard, 1829

##### ﻿ Pupina aureola

Taxon classificationAnimaliaGastropodaPupinidae

Stoliczka, 1872

AF8E6001-549E-57E7-B7D3-7F0DC7563377

Figs 7A , 19E

###### Material examined.

Malaysia • Penang Hill, Moon Gate Station 5 trail, Plot.10; 5.4305°N, 100.2913°E; 250 m a.s.l.; 7 Sept. 2022; T.S. Liew, J. Dulipat, S.M. Goh leg.; BOR/MOL 15124 • Penang Hill, Moon Gate Station 5 trail, Plot.11; 5.4341°N, 100.2921°E; 110 m a.s.l.; 7 Sept. 2022; T.S. Liew, J. Dulipat, S.M. Goh leg.; BOR/MOL 15094 • Penang Hill, Trail - Moniot Road East, Plot.28; 5.4240°N, 100.2746°E; 530 m a.s.l.; 26 Oct. 2022; T.S. Liew, J. Dulipat leg.; BOR/MOL 15225.

**Figure 7.**
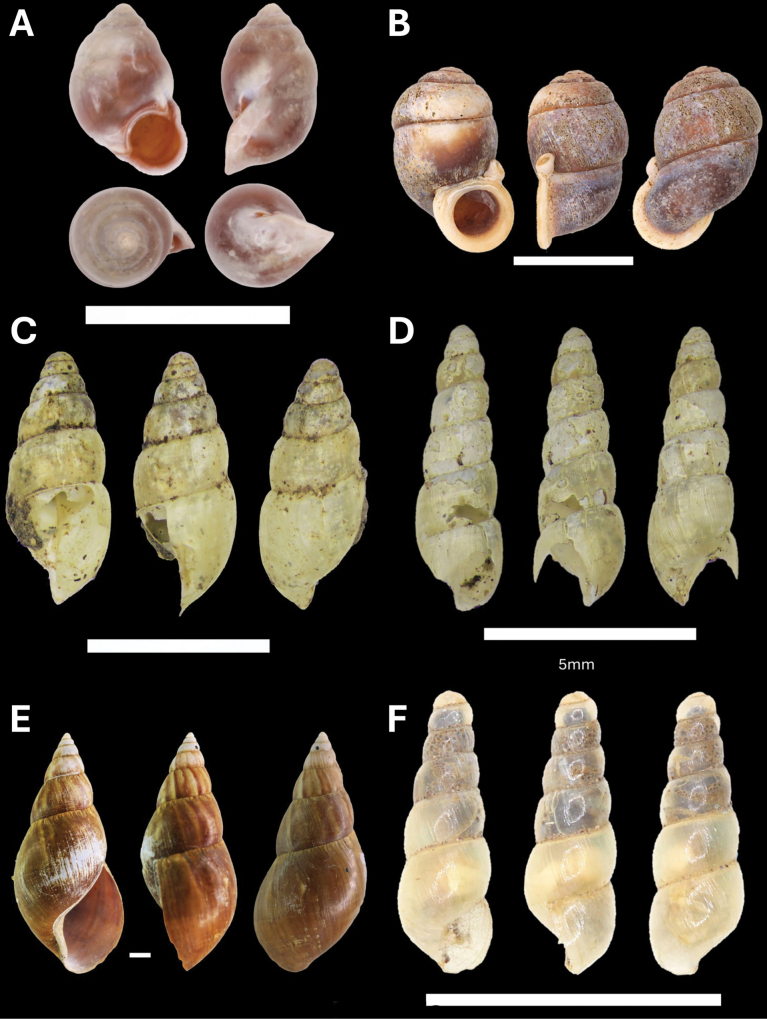
**A***Pupinaaureola* Stoliczka, 1872 BOR/MOL 15225 **B***Rhaphauluslorraini* L. Pfeiffer, 1856 BOR/MOL 15285 **C***Allopeasclavulinum* (Potiez & Michaud, 1838) BOR/MOL 15419 **D***Allopeasgracile* (T. Hutton, 1834) BOR/MOL 15547 **E***Lissachatinafulica* (Bowdich, 1822) BOR/MOL 15195 **F***Subulinaoctona* (Bruguière, 1789) BOR/MOL 15545. Scale bars: 5 mm (**A–C, D, F**); 10 mm (**E**).

###### Remark.

This species is also recorded from the northern part of Peninsular Malaysia and southern Thailand (Maassen 2001; Jirapatrasilp et al. 2022).

#### ﻿Genus *Rhaphaulus* L. Pfeiffer, 1856

##### ﻿ Rhaphaulus lorraini

Taxon classificationAnimaliaGastropodaPupinidae

L. Pfeiffer, 1856

ABBEFC35-FCFB-52AD-88A2-0913BCADC1C0

Figs 7B , 19F

###### Material examined.

Malaysia • Penang Hill, Trail to Western hill, Km 3.6, Plot.23; 5.4203°N, 100.2517°E; 750 m a.s.l.; 24 Oct. 2022; T.S. Liew, J. Dulipat, M.Y. Lee leg.; BOR/MOL 15226 • same data as for preceding; BOR/MOL 15285.

###### Remarks.

This species was described from Penang Hill (Pfeiffer 1856) but was not recorded by Stoliczka (1872, 1873). It is a widespread species in Malaysia and southern Thailand (Laidlaw 1928; Pall-Gergely et al. 2014; Jirapatrasilp et al. 2022).

#### ﻿Subclass Heterobranchia

**Informal group Pulmonata Cuvier, in de Blainville 1814**

**Superfamily Achatinoidea Swainson, 1840**

**Family Achatinidae Swainson, 1840**

**Genus *Allopeas* H. B. Baker, 1935**

##### ﻿ Allopeas clavulinum

Taxon classificationAnimaliaGastropodaAchatinidae

(Potiez & Michaud, 1838)

B0966550-CC45-5A5A-860F-36FB59882E5A

Fig. 7C

###### Material examined.

Malaysia • Penang Hill, The Habitat, Habitat nature trail, Plot.05; 5.4228°N, 100.2653°E; 770 m a.s.l.; 5 Sept. 2022; T.S. Liew, J. Dulipat, S.M. Goh leg.; BOR/MOL 15416 • Penang Hill, The Habitat, Habitat nature trail, Plot.07; 5.4210°N, 100.2642°E; 760 m a.s.l.; 6 Sept. 2022; T.S. Liew, J. Dulipat, S.M. Goh, M.Y. Lee leg.; BOR/MOL 15418 • Penang Hill, Moon Gate Station 5 trail, Plot.11; 5.4341°N, 100.2921°E; 110 m a.s.l.; 7 Sept. 2022; T.S. Liew, J. Dulipat, S.M. Goh leg.; BOR/MOL 15420 • Penang Hill, Teluk Bahang-Balik Pulau, Entrance of a durian farm, near Boulder Valley, Old rubber tree, Plot.17; 5.418°N, 100.2159°E; 180 m a.s.l.; 22 Oct. 2022; T.S. Liew, J. Dulipat leg.; BOR/MOL 15415 • Penang Hill, Teluk Bahang- Balik Pulau, Lam durian farm, Plot.19; 5.4218°N, 100.2263°E; 280 m a.s.l.; 23 Oct. 2022; T.S. Liew, J. Dulipat, M.Y. Lee leg.; BOR/MOL 15419 • same data as for preceding; BOR/MOL 15421 • Penang Hill, Air Itam, Forest near Dam, Plot.24; 5.3904°N, 100.2612°E; 380 m a.s.l.; 24 Oct. 2022; T.S. Liew, J. Dulipat leg.; BOR/MOL 15417 • Penang Hill, Botanical garden, Plot.29; 5.4400°N, 100.2866°E; 75 m a.s.l.; 2 Feb. 2023; T.S. Liew, M.Y. Lee, A. Yusni leg.; BOR/MOL 15750 • Penang Hill, By Path H, Plot.33; 5.4210°N, 100.2701°E; 610 m a.s.l.; 4 Feb. 2023; T.S. Liew, A. Yusni leg.; BOR/MOL 15751.

###### Remark.

New record for Penang Hill. It is possibly an introduced species and is widespread in Malaysia (Foon et al. 2017).

##### ﻿ Allopeas gracile

Taxon classificationAnimaliaGastropodaAchatinidae

(T. Hutton, 1834)

E6B13B9C-DD00-5850-92B8-C952C2145316

Fig. 7D

###### Material examined.

Malaysia • Penang Hill, The Habitat, Research trail, Plot.06; 5.4247°N, 100.2674°E; 750 m a.s.l.; 5 Sept. 2022; T.S. Liew, J. Dulipat, S.M. Goh, M.Y. Lee, M.Y. Wua leg.; BOR/MOL 15414 • Penang Hill, Teluk Bahang- Balik Pulau, Lam durian farm, Plot.13; 5.4198°N, 100.2259°E; 290 m a.s.l.; 21 Oct. 2022; T.S. Liew, J. Dulipat, M.Y. Lee leg.; BOR/MOL 15546 • Penang Hill, Teluk Bahang- Balik Pulau, Tropical fruit farm, Plot.14; 5.4148°N, 100.2187°E; 260 m a.s.l.; 21 Oct. 2022; T.S. Liew, J. Dulipat, M.Y. Lee leg.; BOR/MOL 15547 • Penang Hill, Teluk Bahang- Balik Pulau, Tropical fruit farm, Plot.15; 5.4160°N, 100.2200°E; 250 m a.s.l.; 21 Oct. 2022; T.S. Liew, J. Dulipat, M.Y. Lee leg.; BOR/MOL 15548 • Penang Hill, Teluk Bahang- Balik Pulau, Lam durian farm, Plot.19; 5.4218°N, 100.2263°E; 280 m a.s.l.; 23 Oct. 2022; T.S. Liew, J. Dulipat, M.Y. Lee leg.; BOR/MOL 15284 • Penang Hill, Botanical garden, Plot.29; 5.4400°N, 100.2866°E; 75 m a.s.l.; 2 Feb. 2023; T.S. Liew, M.Y. Lee, A. Yusni leg.; BOR/MOL 15749.

###### Remarks.

More common in disturbed habitats on Penang Hill. It is possibly an introduced species and is widespread in Malaysia (Foon et al. 2017).

#### ﻿Genus *Lissachatina* Bequaert, 1950

##### ﻿ Lissachatina fulica

Taxon classificationAnimaliaGastropodaAchatinidae

(Bowdich, 1822)

721EBD42-328D-5A6D-B72D-E1C1125D8FFB

Figs 7E , 20A

###### Material examined.

Malaysia • Penang Hill, The Habitat, Habitat nature trail, Plot.04; 5.4231°N, 100.2668°E; 770 m a.s.l.; 5 Sept. 2022; T.S. Liew, J. Dulipat, S.M. Goh leg.; BOR/MOL 15486 • Penang Hill, The Habitat, Habitat nature trail, Plot.05; 5.4228°N, 100.2653°E; 770 m a.s.l.; 5 Sept. 2022; T.S. Liew, J. Dulipat, S.M. Goh leg.; BOR/MOL 15131 • same data as for preceding; BOR/MOL 15195 • Penang Hill, The Habitat, Habitat nature trail, Plot.07; 5.4210°N, 100.2642°E; 760 m a.s.l.; 6 Sept. 2022; T.S. Liew, J. Dulipat, S.M. Goh, M.Y. Lee leg.; BOR/MOL 15088 • same data as for preceding; MOL 15147 • same data as for preceding; BOR/MOL 15423 • Penang Hill, Teluk Bahang- Balik Pulau, Lam durian farm, Plot.13; 5.4198°N, 100.2259°E; 290 m a.s.l.; 21 Oct. 2022; T.S. Liew, J. Dulipat, M.Y. Lee leg.; BOR/MOL 15271 • Penang Hill, Teluk Bahang- Balik Pulau, Tropical fruit farm, Plot.15; 5.4160°N, 100.2200°E; 250 m a.s.l.; 21 Oct. 2022; T.S. Liew, J. Dulipat, M.Y. Lee leg.; BOR/MOL 15272 • Penang Hill, Teluk Bahang-Balik Pulau, Entrance of a durian farm, near Boulder Valley, Old rubber tree, Plot.17; 5.418°N, 100.2159°E; 180 m a.s.l.; 22 Oct. 2022; T.S. Liew, J. Dulipat leg.; BOR/MOL 15412 • Penang Hill, Teluk Bahang- Balik Pulau, Lam durian farm, Plot.19; 5.4218°N, 100.2263°E; 280 m a.s.l.; 23 Oct. 2022; T.S. Liew, J. Dulipat, M.Y. Lee leg.; BOR/MOL 15230 • same data as for preceding; BOR/MOL 15283 • Penang Hill, Botanical garden, Plot.29; 5.4400°N, 100.2866°E; 75 m a.s.l.; 2 Feb. 2023; T.S. Liew, M.Y. Lee, A. Yusni leg.; BOR/MOL 15731 • Penang Hill, By Path H, Plot.33; 5.4210°N, 100.2701°E; 610 m a.s.l.; 4 Feb. 2023; T.S. Liew, A. Yusni leg.; BOR/MOL 15727.

###### Remarks.

New record for Penang Hill. This species was likely introduced to Malaysia as earlier as 1911 in Kedah, and 1922 in Penang (South 1926; Berry and Chan 1968). A widespread introduced species in Malaysia.

#### ﻿Genus *Paropeas* Pilsbry, 1906

##### ﻿ Paropeas tchehelense

Taxon classificationAnimaliaGastropodaAchatinidae

(de Morgan, 1885)

4BEE8EC6-610F-55B2-838D-66F373798297

Fig. 8B

###### Material examined.

Malaysia • Penang Hill, The Habitat, Research trail, Plot.01; 5.4248°N, 100.2669°E; 720 m a.s.l.; 5 Sept. 2022; T.S. Liew, J. Dulipat, S.M. Goh leg.; BOR/MOL 15162 • Penang Hill, The Habitat, Habitat nature trail, Plot.08; 5.4223°N, 100.2648°E; 760 m a.s.l.; 6 Sept. 2022; J. Dulipat, S.M. Goh, M.Y. Lee leg.; BOR/MOL 15097.

**Figure 8.**
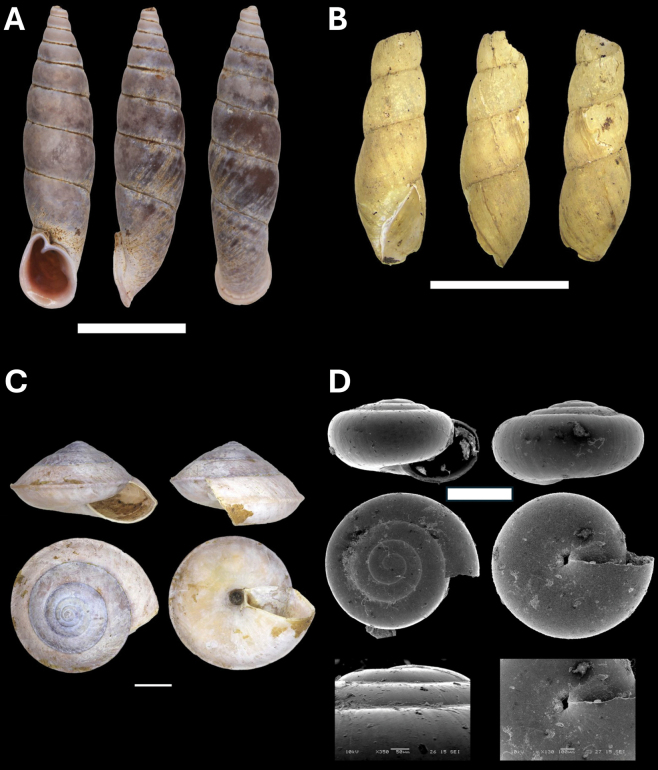
**A***Oospirapenangensis* (Stoliczka, 1873) BOR/MOL 15243 **B***Prosopeastchehelense* (de Morgan, 1885) BOR/MOL 15162 **C***Hemiplectacymatium* (L. Pfeiffer, 1856) BOR/MOL 15736 **D***Microcystina* Penang sp. 1 BOR/MOL 15754. Scale bars: 10 mm (**A–C**); 0.5 mm (**D**).

###### Remark.

New record from Penang Hill. This species is also recorded from the mainland of Peninsular Malaysia and southern Thailand (Maassen 2001; Foon et al. 2017).

#### ﻿Genus *Subulina* H. Beck, 1837

##### ﻿ Subulina octona

Taxon classificationAnimaliaGastropodaAchatinidae

(Bruguière, 1789)

6A4FBFE0-863C-5345-B177-B31BA40C36C7

Fig. 7F

###### Material examined.

Malaysia • Penang Hill, Teluk Bahang- Balik Pulau, Lam durian farm, Plot.19; 5.4218°N, 100.2263°E; 280 m a.s.l.; 23 Oct. 2022; T.S. Liew, J. Dulipat, M.Y. Lee leg.; BOR/MOL 15422 • Penang Hill, Trail to Western hill, Junction to Teluk Bahang from Penang Hill, Plot.22; 5.4223°N, 100.2494°E; 760 m a.s.l.; 24 Oct. 2022; T.S. Liew, J, Dulipat, M.Y. Lee leg.; BOR/MOL 15545 • Penang Hill, Botanical garden, Plot.29; 5.4400°N, 100.2866°E; 75 m a.s.l.; 2 Feb. 2023; T.S. Liew, M.Y. Lee, A. Yusni leg.; BOR/MOL 15748.

###### Remarks.

New record from Penang Hill. This species is also recorded from the mainland of Peninsular Malaysia (Maassen 2001; Foon et al. 2017).

#### ﻿Superfamily Arionoidea Gray, 1840

**Family Philomycidae Gray, 1847**

**Genus *Meghimatium* van Hasselt, 1823**

##### ﻿ Meghimatium pictum

Taxon classificationAnimaliaGastropodaPhilomycidae

(Stoliczka, 1873)

BB4B3413-6722-5281-B428-C8A1BD2CFB05

Fig. 22E

###### Remarks.

This species was previously described by Stoliczka (1873) from the base of the hill in the north, at ca 100 feet above sea level. It was not found during the sampling of this study. However, this species was recently photographed on Penang Hill (Fig. 22E). It is thought to be a widespread species found from East Asia to Indochina ([Bibr B50][Bibr B22]; Ito et al. 2023).

#### ﻿Superfamily Clausilioidea J. E. Gray, 1855

**Family Clausiliidae J. E. Gray, 1855**

**Genus *Oospira* W. T. Blanford, 1872**

##### ﻿ Oospira penangensis

Taxon classificationAnimaliaGastropodaClausiliidae

(Stoliczka, 1873)

79CE6989-3838-526A-80D9-02FCE267FF71

Figs 8A , 20B

###### Material examined.

Malaysia • Penang Hill, Air Itam, Forest near Dam, Plot.24; 5.3904°N, 100.2612°E; 380 m a.s.l.; 24 Oct. 2022; T.S. Liew, J. Dulipat leg.; BOR/MOL 15223 • Penang Hill, Air Itam, Forest near Dam, Plot.25; 5.3900°N, 100.2575°E; 290 m a.s.l.; 25 Oct. 2022; T.S. Liew, J. Dulipat leg.; BOR/MOL 15224 • Penang Hill, Trail - Moniot Road East, Plot.28; 5.4240°N, 100.2746°E; 530 m a.s.l.; 26 Oct. 2022; T.S. Liew, J. Dulipat leg.; BOR/MOL 15243.

###### Remark.

This species was described and recorded by Stoliczka (1873) from Penang Hill, and it can also be found in Perak (Maassen 2001).

#### ﻿Genus *Phaedusa* H. Adams & A. Adams, 1855

##### ﻿ Phaedusa filicostata

Taxon classificationAnimaliaGastropodaClausiliidae

(Stoliczka, 1873)

7D7F43FF-C866-5EC6-89A1-13123E32AEE2

###### Remark.

Previously described and recorded by Stoliczka (1873) from Penang Hill but not found in this study.

#### ﻿Superfamily Helicarionoidea Bourguignat, 1877

**Family Ariophantidae Godwin-Austen, 1883**

**Genus *Hemiplecta* Albers, 1850**

##### ﻿ Hemiplecta cymatium

Taxon classificationAnimaliaGastropodaAriophantidae

(L. Pfeiffer, 1856)

447C3480-8AE2-51E4-B2E4-48934E05DD94

Figs 8C , 20C

###### Material examined.

Malaysia • Penang Hill, The Habitat, Research trail, Plot.01; 5.4248°N, 100.2669°E; 720 m a.s.l.; 5 Sept. 2022; T.S. Liew, J. Dulipat, S.M. Goh leg.; BOR/MOL 15161 • Penang Hill, The Habitat, Research trail, Plot.02; 5.4237°N, 100.2649°E; 720 m a.s.l.; 5 Sept. 2022; T.S. Liew, J. Dulipat, S.M. Goh leg.; BOR/MOL 15158 • Penang Hill, The Habitat, Habitat nature trail, Plot.05; 5.4228°N, 100.2653°E; 770 m a.s.l.; 5 Sept. 2022; T.S. Liew, J. Dulipat, S.M. Goh leg.; BOR/MOL 15136 • Penang Hill, The Habitat, Research trail, Plot.06; 5.4247°N, 100.2674°E; 750 m a.s.l.; 5 Sept. 2022; T.S. Liew, J. Dulipat, S.M. Goh, M.Y. Lee, M.Y. Wua leg.; BOR/MOL 15137 • Penang Hill, The Habitat, Habitat nature trail, Plot.07; 5.4210°N, 100.2642°E; 760 m a.s.l.; 6 Sept. 2022; T.S. Liew, J. Dulipat, S.M. Goh, M.Y. Lee leg.; BOR/MOL 15087 • same data as for preceding; BOR/MOL 15105 • same data as for preceding; BOR/MOL 15142 • Penang Hill, The Habitat, Habitat nature trail, Plot.08; 5.4223°N, 100.2648°E; 760 m a.s.l.; 6 Sept. 2022; J. Dulipat, S.M. Goh, M.Y. Lee leg.; BOR/MOL 15096 • Penang Hill, Botanical garden, Plot.12; 5.4396°N, 100.2860°E; 85 m a.s.l.; 4 Sept. 2022; T.S. Liew, J. Dulipat, S.M. Goh leg.; BOR/MOL 15092 • Penang Hill, Teluk Bahang, Taman Rimba Teluk Bahang, Trail, Simpang 6, Plot.16; 5.4429°N, 100.2212°E; 120 m a.s.l.; 21 Oct. 2022; T.S. Liew, J. Dulipat, M.Y. Lee leg.; BOR/MOL 15269 • Penang Hill, Teluk Bahang-Balik Pulau, Entrance of a durian farm, near Boulder Valley, Old rubber tree, Plot.17; 5.418°N, 100.2159°E; 180 m a.s.l.; 22 Oct. 2022; T.S. Liew, J. Dulipat leg.; BOR/MOL 15277 • Penang Hill, Air Itam, Forest near Dam, Plot.24; 5.3904°N, 100.2612°E; 380 m a.s.l.; 24 Oct. 2022; T.S. Liew, J. Dulipat leg.; BOR/MOL 15220 • same data as for preceding; BOR/MOL 15290 • Penang Hill, Air Itam, Forest near Dam, Plot.25; 5.3900°N, 100.2575°E; 290 m a.s.l.; 25 Oct. 2022; T.S. Liew, J. Dulipat leg.; BOR/MOL 15294 • Penang Hill, Botanical garden, Plot.29; 5.4400°N, 100.2866°E; 75 m a.s.l.; 2 Feb. 2023; T.S. Liew, M.Y. Lee, A. Yusni leg.; BOR/MOL 15736 • Penang Hill, By Path H, Plot.33; 5.4210°N, 100.2701°E; 610 m a.s.l.; 4 Feb. 2023; T.S. Liew, A. Yusni leg.; BOR/MOL 15735 • Penang Hill, Path B, Random1. PathB; 5.4216°N, 100.2674°E; 730 m a.s.l.; 4 Sept. 2022; T.S. Liew, J. Dulipat leg.; BOR/MOL 15104 • same data as for preceding; BOR/MOL 15148.

###### Remarks.

One of the most common large land snails at Penang Hill, with a shell width of up to 40 mm. It is also known from other localities in Peninsular Malaysia. This species can also be found in Perak (Maassen 2001; Foon et al. 2017).

#### ﻿Genus *Microcystina* Mörch, 1872

##### ﻿ Microcystina

Taxon classificationAnimaliaGastropodaAriophantidae

Penang sp. 1

D8CCB3B5-E746-5A61-89D1-BDA7EA15FC65

Fig. 8D

###### Material examined.

Malaysia • Penang Hill, The Habitat, Habitat nature trail, Plot.05; 5.4228°N, 100.2653°E; 770 m a.s.l.; 5 Sept. 2022; T.S. Liew, J. Dulipat, S.M. Goh leg.; BOR/MOL 15453 • Penang Hill, The Habitat, Research trail, Plot.06; 5.4247°N, 100.2674°E; 750 m a.s.l.; 5 Sept. 2022; T.S. Liew, J. Dulipat, S.M. Goh, M.Y. Lee, M.Y. Wua leg.; BOR/MOL 15455 • Penang Hill, The Habitat, Habitat nature trail, Plot.07; 5.4210°N, 100.2642°E; 760 m a.s.l.; 6 Sept. 2022; T.S. Liew, J. Dulipat, S.M. Goh, M.Y. Lee leg.; BOR/MOL 15448 • Penang Hill, The Habitat, Habitat nature trail, Plot.08; 5.4223°N, 100.2648°E; 760 m a.s.l.; 6 Sept. 2022; J. Dulipat, S.M. Goh, M.Y. Lee leg.; BOR/MOL 15450 • Penang Hill, Moon Gate Station 5 trail, Plot.09; 5.4216°N, 100.2857°E; 410 m a.s.l.; 7 Sept. 2022; T.S. Liew, J. Dulipat, S.M. Goh leg.; BOR/MOL 15445 • Penang Hill, Moon Gate Station 5 trail, Plot.10; 5.4305°N, 100.2913°E; 250 m a.s.l.; 7 Sept. 2022; T.S. Liew, J. Dulipat, S.M. Goh leg.; BOR/MOL 15451 • Penang Hill, Moon Gate Station 5 trail, Plot.11; 5.4341°N, 100.2921°E; 110 m a.s.l.; 7 Sept. 2022; T.S. Liew, J. Dulipat, S.M. Goh leg.; BOR/MOL 15449 • Penang Hill, Teluk Bahang- Balik Pulau, Lam durian farm, Plot.13; 5.4198°N, 100.2259°E; 290 m a.s.l.; 21 Oct. 2022; T.S. Liew, J. Dulipat, M.Y. Lee leg.; BOR/MOL 15558 • Penang Hill, Teluk Bahang, Taman Rimba Teluk Bahang, Trail, Simpang 6, Plot.16; 5.4429°N, 100.2212°E; 120 m a.s.l.; 21 Oct. 2022; T.S. Liew, J. Dulipat, M.Y. Lee leg.; BOR/MOL 15557 • Penang Hill, Teluk Bahang-Balik Pulau, Entrance of a durian farm, near Boulder Valley, Old rubber tree, Plot.17; 5.418°N, 100.2159°E; 180 m a.s.l.; 22 Oct. 2022; T.S. Liew, J. Dulipat leg.; BOR/MOL 15447 • Penang Hill, Teluk Bahang, Taman Rimba Teluk Bahang, Trail Stesen 3, Plot.18; 5.4420°N, 100.2218°E; 150 m a.s.l.; 22 Oct. 2022; T.S. Liew, J. Dulipat leg.; BOR/MOL 15452 • Penang Hill, Air Itam, Forest near farm, Plot.21; 5.3908°N, 100.2637°E; 340 m a.s.l.; 23 Oct. 2022; T.S. Liew, J. Dulipat leg.; BOR/MOL 15555 • Penang Hill, Trail to Western hill, Junction to Teluk Bahang from Penang Hill, Plot.22; 5.4223°N, 100.2494°E; 760 m a.s.l.; 24 Oct. 2022; T.S. Liew, J, Dulipat, M.Y. Lee leg.; BOR/MOL 15556 • Penang Hill, Trail to Western hill, Km 3.6, Plot.23; 5.4203°N, 100.2517°E; 750 m a.s.l.; 24 Oct. 2022; T.S. Liew, J. Dulipat, M.Y. Lee leg.; BOR/MOL 15554 • Penang Hill, Air Itam, Forest near Dam, Plot.26; 5.3977°N, 100.2612°E; 290 m a.s.l.; 25 Oct. 2022; T.S. Liew, J. Dulipat leg.; BOR/MOL 15454 • Penang Hill, Trail - Moniot Road East, Plot.28; 5.4240°N, 100.2746°E; 530 m a.s.l.; 26 Oct. 2022; T.S. Liew, J. Dulipat leg.; BOR/MOL 15446 • Penang Hill, By Path H, Plot.33; 5.4210°N, 100.2701°E; 610 m a.s.l.; 4 Feb. 2023; T.S. Liew, A. Yusni leg.; BOR/MOL 15754.

###### Remarks.

This is a new record for Penang Hill of a widespread species. The shell has a slightly raised spire with a height less than half of the last whorl height. The upper shell, including the apical and subsequent whorls, shows growth lines, while the lower shell exhibits faint spiral lines.

##### ﻿ Microcystina

Taxon classificationAnimaliaGastropodaAriophantidae

Penang sp. 2

F7B4952B-3B25-5B0A-BCE9-1EC73901F42C

Fig. 9A

###### Material examined.

Malaysia • Penang Hill, The Habitat, Research trail, Plot.01; 5.4248°N, 100.2669°E; 720 m a.s.l.; 5 Sept. 2022; T.S. Liew, J. Dulipat, S.M. Goh leg.; BOR/MOL 15456 • Penang Hill, The Habitat, Habitat nature trail, Plot.07; 5.4210°N, 100.2642°E; 760 m a.s.l.; 6 Sept. 2022; T.S. Liew, J. Dulipat, S.M. Goh, M.Y. Lee leg.; BOR/MOL 15458 • Penang Hill, Air Itam, Forest near Dam, Plot.25; 5.3900°N, 100.2575°E; 290 m a.s.l.; 25 Oct. 2022; T.S. Liew, J. Dulipat leg.; BOR/MOL 15459 • Penang Hill, Trail - Moniot Road East, Plot.28; 5.4240°N, 100.2746°E; 530 m a.s.l.; 26 Oct. 2022; T.S. Liew, J. Dulipat leg.; BOR/MOL 15457 • Penang Hill, By Path H, Plot.33; 5.4210°N, 100.2701°E; 610 m a.s.l.; 4 Feb. 2023; T.S. Liew, A. Yusni leg.; BOR/MOL 15763.

**Figure 9.**
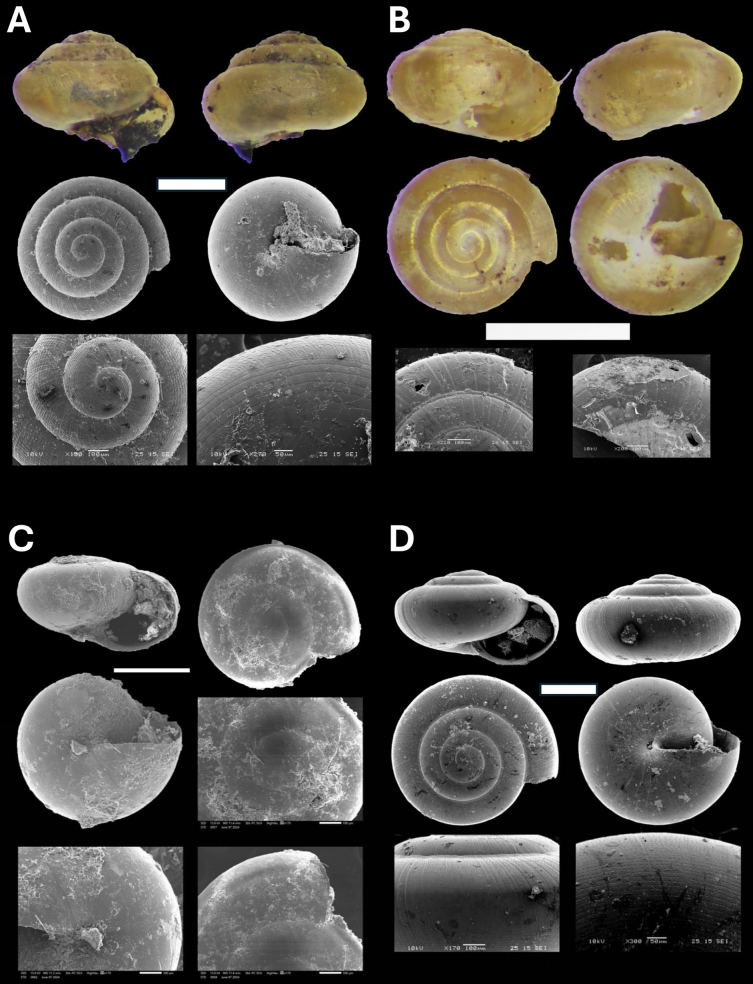
**A***Microcystina* Penang sp. 2 BOR/MOL 15458 **B***Microcystina* Penang sp. 4 BOR/MOL 15755 **C***Microcystina* Penang sp. 5 BOR/MOL 15762 **D***Microcystina* Penang sp. 6 BOR/MOL 15756. Scale bar: 0.5 mm.

###### Remarks.

This is a new record for Penang Hill. The shell spire is highly raised, with a height more than half of the last whorl height. This species has the most convex shell compared to other *Microcystina* species from Penang Hill. On the upper shell, the apical whorls have elevated spiral ribs that continue onto subsequent whorls, which are crossed by radial ribs forming granule-like sculptures. Below the shell, there are spaced grooves with spiral lines at the periphery, which reduce towards the umbilicus.

##### ﻿ Microcystina

Taxon classificationAnimaliaGastropodaAriophantidae

Penang sp. 4

45B38426-0F6C-5804-BE8F-8BB777AB9739

Fig. 9B

###### Material examined.

Malaysia • Penang Hill, The Habitat, Research trail, Plot.03; 5.4226°N, 100.2645°E; 750 m a.s.l.; 5 Sept. 2022; T.S. Liew, J. Dulipat, S.M. Goh leg.; BOR/MOL 15462 • Penang Hill, Botanical garden, Plot.12; 5.4396°N, 100.2860°E; 85 m a.s.l.; 4 Sept. 2022; T.S. Liew, J. Dulipat, S.M. Goh leg.; BOR/MOL 15463 • Penang Hill, Moniot trail, Plot.32; 5.4165°N, 100.2574°E; 695 m a.s.l.; 4 Feb. 2023; T.S. Liew, A. Yusni leg.;BOR/MOL 15755.

###### Remarks.

This is a new record for Penang Hill. The shell spire is moderately raised, with a height less than half of the last whorl height. On the upper shell, the apical whorls are smooth, and subsequent whorls have regularly spaced deep radial grooves. This species has the most prominent radial grooves compared to other *Microcystina* species from Penang Hill. Below the shell, there are regularly spaced, deep, radial grooves that continue from the upper shell.

##### ﻿ Microcystina

Taxon classificationAnimaliaGastropodaAriophantidae

Penang sp. 5

58EA9368-F879-52DA-8C2C-4FEDA56DD2BC

Fig. 9C

###### Material examined.

Malaysia • Penang Hill, Air Itam, Forest near Dam, Plot.26; 5.3977°N, 100.2612°E; 290 m a.s.l.; 25 Oct. 2022; T.S. Liew, J. Dulipat leg.; BOR/MOL 15464 • Penang Hill, By Path H, Plot.33; 5.4210°N, 100.2701°E; 610 m a.s.l.; 4 Feb. 2023; T.S. Liew, A. Yusni leg.; BOR/MOL 15762.

###### Remarks.

This is a new record for Penang Hill. The shell spire is hardly raised, with a height less than half of the last whorl height. On the upper shell, there are densely spirally arranged pits on the apical and subsequent whorls. This species is unique among the *Microcystina* species from Penang Hill in having densely spirally arranged pits on the whorls near the periphery. Below the shell, there are densely spirally arranged pits that continue from the upper shell and fade away towards the umbilicus.

##### ﻿ Microcystina

Taxon classificationAnimaliaGastropodaAriophantidae

Penang sp. 6

CA378F8A-4798-5D02-9FD0-2BBB84B10A9B

Fig. 9D

###### Material examined.

Malaysia • Penang Hill, Teluk Bahang- Balik Pulau, Lam durian farm, Plot.13; 5.4198°N, 100.2259°E; 290 m a.s.l.; 21 Oct. 2022; T.S. Liew, J. Dulipat, M.Y. Lee leg.; BOR/MOL 15559 • Penang Hill, Botanical garden, Plot.29; 5.4400°N, 100.2866°E; 75 m a.s.l.; 2 Feb. 2023; T.S. Liew, M.Y. Lee, A. Yusni leg.; BOR/MOL 15756.

###### Remarks.

This is a new record for Penang Hill. The shell spire is slightly raised, and the shell whorls are more rapidly expanded compared to other *Microcystina* species from Penang Hill. On the upper shell, there are densely spirally arranged pits on the apical whorls, transitioning to densely placed spiral grooves on the subsequent whorls, which become more densely placed towards the periphery. Below the shell, there are densely placed spiral grooves that continue from the upper shell and fade away towards the umbilicus.

#### ﻿Genus *Parmarion* P. Fischer, 1855

##### ﻿ Parmarion martensi

Taxon classificationAnimaliaGastropodaAriophantidae

Simroth, 1893

C8675D6B-57F5-5DF2-9FAA-307D4DCE2827

Figs 10A , 20D

###### Material examined.

Malaysia • Penang Hill, Teluk Bahang- Balik Pulau, Tropical fruit farm, Plot.15; 5.4160°N, 100.2200°E; 250 m a.s.l.; 21 Oct. 2022; T.S. Liew, J. Dulipat, M.Y. Lee leg.; BOR/MOL 15229 • same data as for preceding; BOR/MOL 15239.

**Figure 10.**
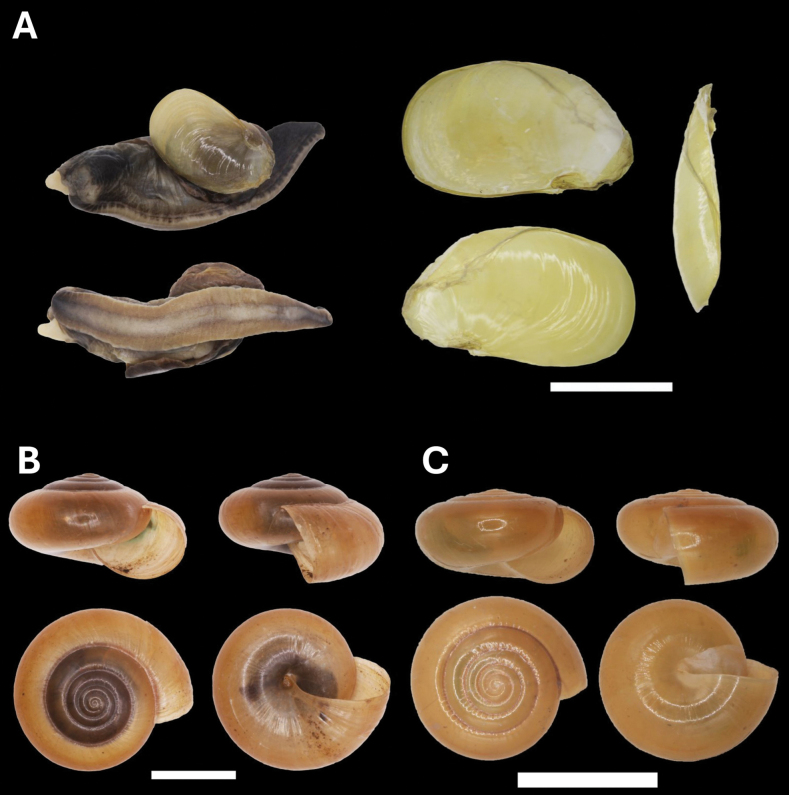
**A***Parmarionmartensi* Simroth, 1893 BOR/MOL 15239 **B**Tanychlamysindica (Godwin Austen, 1883) BOR/MOL 15771 **C***Tanychlamys* Penang sp. 1 BOR/MOL 15151. Scale bar: 10 mm.

###### Remark.

This is a new record for Penang Hill. This species is widespread in Malaysia and might have been introduced to this location (Maassen 2001).

#### ﻿Genus *Tanychlamys* W. H. Benson, 1834

##### ﻿ Tanychlamys indica

Taxon classificationAnimaliaGastropodaAriophantidae

(Godwin Austen, 1883)

D721DBF7-02FB-556D-8DE7-623627CA8C40

Figs 10B , 20E

###### Material examined.

Malaysia • Penang Hill, Along Jalan Tunku Yahya Petra, Random6.; 5.4225°N, 100.2662°E; 730 m a.s.l.; 20 Jun. 2023; T.S. Liew leg.; BOR/MOL 15771.

###### Remarks.

This is a new record for Penang Hill. This species is widespread in Malaysia and might have been introduced to this location (Maassen 2001).

##### ﻿ Tanychlamys

Taxon classificationAnimaliaGastropodaAriophantidae

Penang sp. 1

4BA934A2-EE63-517E-BF63-86846F7BAB60

Figs 10C , 20F

###### Material examined.

Malaysia • Penang Hill, The Habitat, Research trail, Plot.01; 5.4248°N, 100.2669°E; 720 m a.s.l.; 5 Sept. 2022; T.S. Liew, J. Dulipat, S.M. Goh leg.; BOR/MOL 15160 • Penang Hill, The Habitat, Research trail, Plot.03; 5.4226°N, 100.2645°E; 750 m a.s.l.; 5 Sept. 2022; T.S. Liew, J. Dulipat, S.M. Goh leg.;, BOR/MOL 15198 • Penang Hill, The Habitat, Habitat nature trail, Plot.04; 5.4231°N, 100.2668°E; 770 m a.s.l.; 5 Sept. 2022; T.S. Liew, J. Dulipat, S.M. Goh leg.; BOR/MOL 15130 • Penang Hill, The Habitat, Habitat nature trail, Plot.05; 5.4228°N, 100.2653°E; 770 m a.s.l.; 5 Sept. 2022; T.S. Liew, J. Dulipat, S.M. Goh leg.; BOR/MOL 15132 • Penang Hill, The Habitat, Habitat nature trail, Plot.07; 5.4210°N, 100.2642°E; 760 m a.s.l.; 6 Sept. 2022; T.S. Liew, J. Dulipat, S.M. Goh, M.Y. Lee leg.; BOR/MOL 15145 • Penang Hill, The Habitat, Habitat nature trail, Plot.08; 5.4223°N, 100.2648°E; 760 m a.s.l.; 6 Sept. 2022; J. Dulipat, S.M. Goh, M.Y. Lee leg.; BOR/MOL 15098 • same data as for preceding; BOR/MOL 15154 • Penang Hill, Moon Gate Station 5 trail, Plot.11; 5.4341°N, 100.2921°E; 110 m a.s.l.; 7 Sept. 2022; T.S. Liew, J. Dulipat, S.M. Goh leg.; BOR/MOL 15153 • Penang Hill, Botanical garden, Plot.12; 5.4396°N, 100.2860°E; 85 m a.s.l.; 4 Sept. 2022; T.S. Liew, J. Dulipat, S.M. Goh leg.; BOR/MOL 15413 • Penang Hill, Teluk Bahang- Balik Pulau, Tropical fruit farm, Plot.14; 5.4148°N, 100.2187°E; 260 m a.s.l.; 21 Oct. 2022; T.S. Liew, J. Dulipat, M.Y. Lee leg.; BOR/MOL 15247 • Penang Hill, Teluk Bahang- Balik Pulau, Tropical fruit farm, Plot.15; 5.4160°N, 100.2200°E; 250 m a.s.l.; 21 Oct. 2022; T.S. Liew, J. Dulipat, M.Y. Lee leg.; BOR/MOL 15302 • Penang Hill, Air Itam, Forest near farm, Plot.21; 5.3908°N, 100.2637°E; 340 m a.s.l.; 23 Oct. 2022; T.S. Liew, J. Dulipat leg.; BOR/MOL 15248 • Penang Hill, Trail to Western hill, Km 3.6, Plot.23; 5.4203°N, 100.2517°E; 750 m a.s.l.; 24 Oct. 2022; T.S. Liew, J. Dulipat, M.Y. Lee leg.; BOR/MOL 15549 • Penang Hill, Air Itam, Forest near Dam, Plot.24; 5.3904°N, 100.2612°E; 380 m a.s.l.; 24 Oct. 2022; T.S. Liew, J. Dulipat leg.; BOR/MOL 15244 • same data as for preceding; BOR/MOL 15303 • Penang Hill, Air Itam, Forest near Dam, Plot.25; 5.3900°N, 100.2575°E; 290 m a.s.l.; 25 Oct. 2022; T.S. Liew, J. Dulipat leg.; BOR/MOL 15249 • Penang Hill, Air Itam, Forest near Dam, Plot.26; 5.3977°N, 100.2612°E; 290 m a.s.l.; 25 Oct. 2022; T.S. Liew, J. Dulipat leg.; BOR/MOL 15245 • same data as for preceding; BOR/MOL 15304 • Penang Hill, Trail from Viaduct to Claremont, after shelter, Plot.27; 5.4205°N, 100.2724°E; 500 m a.s.l.; 26 Oct. 2022; T.S. Liew, J. Dulipat leg.; BOR/MOL 15305 • Penang Hill, By Path H, Plot.33; 5.4210°N, 100.2701°E; 610 m a.s.l.; 4 Feb. 2023; T.S. Liew, A. Yusni leg.; BOR/MOL 15740 • Penang Hill, Path B, Random1. PathB; 5.4216°N, 100.2674°E; 730 m a.s.l.; 4 Sept. 2022; T.S. Liew, J. Dulipat leg.; • same data as for preceding; BOR/MOL 15151.

###### Remark.

This is a new record for Penang Hill. It is one of the most common large land snails found there.

##### ﻿ Tanychlamys

Taxon classificationAnimaliaGastropodaAriophantidae

Penang sp. 2

7978E9F0-D9DE-5B00-8FA5-59C8F0D7346D

Figs 11A , 21A

###### Material examined.

Malaysia • Penang Hill, The Habitat, Research trail, Plot.03; 5.4226°N, 100.2645°E; 750 m a.s.l.; 5 Sept. 2022; T.S. Liew, J. Dulipat, S.M. Goh leg.; BOR/MOL 15424 • Penang Hill, Teluk Bahang- Balik Pulau, Bukit Kerajaan ForestReserve next to Lam durian farm, Plot.20; 5.4199°N, 100.2265°E; 330 m a.s.l.; 23 Oct. 2022; T.S. Liew, J. Dulipat, M.Y. Lee leg.; BOR/MOL 15552 • Penang Hill, Air Itam, Forest near farm, Plot.21; 5.3908°N, 100.2637°E; 340 m a.s.l.; 23 Oct. 2022; T.S. Liew, J. Dulipat leg.; BOR/MOL 15550 • Penang Hill, Trail to Western hill, Junction to Teluk Bahang from Penang Hill, Plot.22; 5.4223°N, 100.2494°E; 760 m a.s.l.; 24 Oct. 2022; T.S. Liew, J, Dulipat, M.Y. Lee leg.; BOR/MOL 15551 • Penang Hill, Western hill to Teluk Bahang Trail Stetion 4, Plot.30; 5.4211°N, 100.2420°E; 570 m a.s.l.; 3 Feb. 2023; T.S. Liew, M.Y. Lee, A. Yusni leg.; BOR/MOL 15759 • Penang Hill, Western hill to Teluk Bahang Trail Stetion 7, Plot.31; 5.4291°N, 100.2334°E; 490 m a.s.l.; 3 Feb. 2023; T.S. Liew, M.Y. Lee, A. Yusni leg.; BOR/MOL 15757.

**Figure 11.**
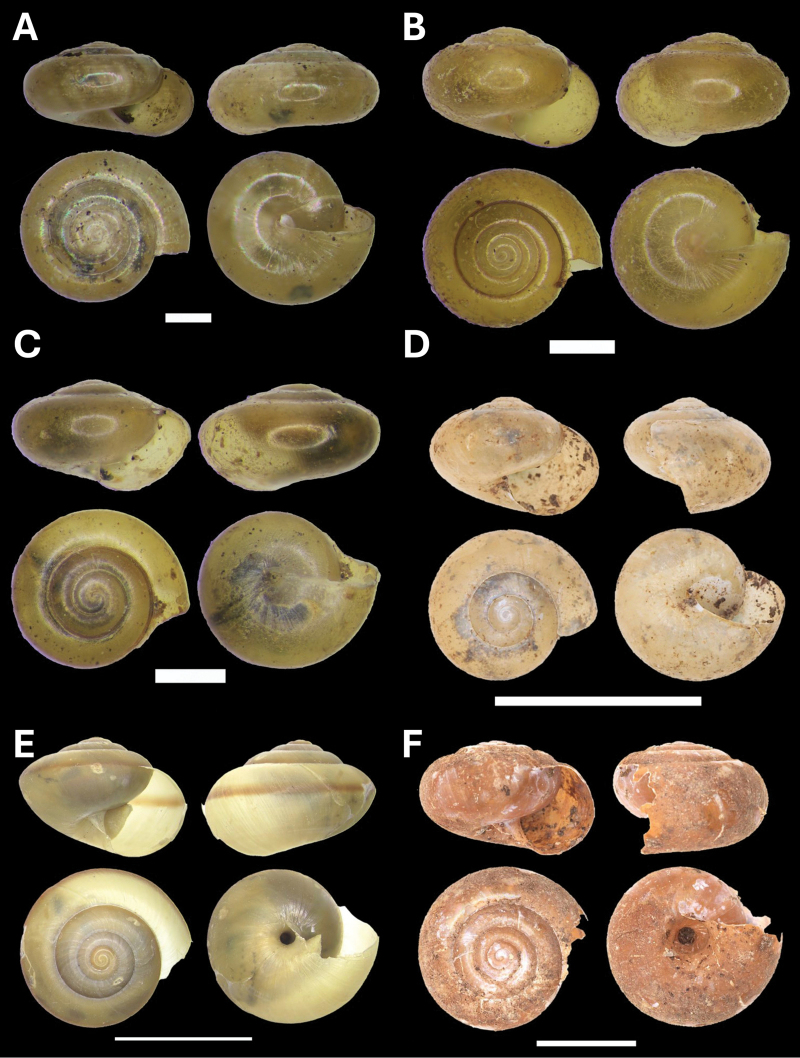
**A***Tanychlamys* Penang sp. 2 BOR/MOL 15759 **B***Tanychlamysstephoides* (Stoliczka, 1873) BOR/MOL 15164 **C***Tanychlamystersa* (Issel, 1874) BOR/MOL 15533 **D***Helicarionpermolle* Stoliczka, 1873 BOR/MOL 15741 **E***Bradybaenasimilaris* (A. Férussac, 1821) BOR/MOL 15772 **F***Trichochloritispenangensis* (Stoliczka, 1873) BOR/MOL 15291. Scale bars: 1 mm (**A–C**); 10 mm (**D–F**).

###### Remark.

This is a new record for Penang Hill.

##### ﻿ Tanychlamys stephoides

Taxon classificationAnimaliaGastropodaAriophantidae

(Stoliczka, 1873)

CA7A7A6D-4609-5FF5-8FDF-A13171375AC4

Fig. 11B

###### Material examined.

Malaysia • Penang Hill, The Habitat, Research trail, Plot.01; 5.4248°N, 100.2669°E; 720 m a.s.l.; 5 Sept. 2022; T.S. Liew, J. Dulipat, S.M. Goh leg.; BOR/MOL 15164.

###### Remark.

This species was previously described by Stoliczka (1873) from Penang Hill.

##### ﻿ Tanychlamys tersa

Taxon classificationAnimaliaGastropodaAriophantidae

(Issel, 1874)

5FE692C9-1D54-5E01-96A5-3004540242E7

Figs 11C , 21B

###### Material examined.

Malaysia • Penang Hill, Teluk Bahang- Balik Pulau, Tropical fruit farm, Plot.14; 5.4148°N, 100.2187°E; 260 m a.s.l.; 21 Oct. 2022; T.S. Liew, J. Dulipat, M.Y. Lee leg.; BOR/MOL 15553 • Penang Hill, Teluk Bahang- Balik Pulau, Tropical fruit farm, Plot.15; 5.4160°N, 100.2200°E; 250 m a.s.l.; 21 Oct. 2022; T.S. Liew, J. Dulipat, M.Y. Lee leg.; BOR/MOL 15246 • Penang Hill, Air Itam, Forest near Dam, Plot.24; 5.3904°N, 100.2612°E; 380 m a.s.l.; 24 Oct. 2022; T.S. Liew, J. Dulipat leg.; BOR/MOL 15250 • Penang Hill, Botanical garden, Plot.29; 5.4400°N, 100.2866°E; 75 m a.s.l.; 2 Feb. 2023; T.S. Liew, M.Y. Lee, A. Yusni leg.; BOR/MOL 15758.

###### Remarks.

This is a new record for Penang Hill. This species is widespread in Malaysia and might have been introduced to this location (Maassen 2001).

#### ﻿Family Helicarionidae Bourguignat, 1877

**Genus *Helicarion* A. Férussac, 1821**

##### ﻿ Helicarion permolle

Taxon classificationAnimaliaGastropodaHelicarionidae

Stoliczka, 1873

BA505C82-5B74-5084-AAF2-75A2C6081E18

Figs 11D , 21C

###### Material examined.

Malaysia • Penang Hill, The Habitat, Habitat nature trail, Plot.04; 5.4231°N, 100.2668°E; 770 m a.s.l.; 5 Sept. 2022; T.S. Liew, J. Dulipat, S.M. Goh leg.; BOR/MOL 15117 • same data as for preceding; BOR/MOL 15480 • Penang Hill, The Habitat, Habitat nature trail, Plot.05; 5.4228°N, 100.2653°E; 770 m a.s.l.; 5 Sept. 2022; T.S. Liew, J. Dulipat, S.M. Goh leg.; BOR/MOL 15196 • Penang Hill, The Habitat, Habitat nature trail, Plot.07; 5.4210°N, 100.2642°E; 760 m a.s.l.; 6 Sept. 2022; T.S. Liew, J. Dulipat, S.M. Goh, M.Y. Lee leg.; BOR/MOL 15144 • Penang Hill, Botanical garden, Plot.12; 5.4396°N, 100.2860°E; 85 m a.s.l.; 4 Sept. 2022; T.S. Liew, J. Dulipat, S.M. Goh leg.; BOR/MOL 15121 • Penang Hill, Trail from Viaduct to Claremont, after shelter, Plot.27; 5.4205°N, 100.2724°E; 500 m a.s.l.; 26 Oct. 2022; T.S. Liew, J. Dulipat leg.; BOR/MOL 15235 • Penang Hill, Botanical garden, Plot.29; 5.4400°N, 100.2866°E; 75 m a.s.l.; 2 Feb. 2023; T.S. Liew, M.Y. Lee, A. Yusni leg.; BOR/MOL 15742 • same data as for preceding; BOR/MOL 15768 • Penang Hill, By Path H, Plot.33; 5.4210°N, 100.2701°E; 610 m a.s.l.; 4 Feb. 2023; T.S. Liew, A. Yusni leg.; BOR/MOL 15741 • Penang Hill, Path B, Random1. PathB; 5.4216°N, 100.2674°E; 730 m a.s.l.; 4 Sept. 2022; T.S. Liew, J. Dulipat leg.; BOR/MOL 15109.

###### Remarks.

This species was previously described by Stoliczka (1873), and it is one of the most common semi-slugs from Penang Hill. It was listed as taxon inquirendum on MolluscaBase (2024) [https://molluscabase.org/aphia.php?p=taxdetails&id=1337679]. We suggest that this is a valid species as the specimens fit the description in Stoliczka (1873).

#### ﻿Superfamily Helicoidea Rafinesque, 1815

**Family Camaenidae Pilsbry, 1895**

**Genus *Amphidromus* Albers, 1850**

##### ﻿ Amphidromus atricallosus

Taxon classificationAnimaliaGastropodaCamaenidae

(A. Gould, 1843)

E99DB71D-225F-5836-9C29-A61E47C5C572

###### Remark.

This species was previously described by Stoliczka (1873) from Penang Hill but was not found during the sampling of this study.

##### ﻿ Amphidromus perversus

Taxon classificationAnimaliaGastropodaCamaenidae

(Linnaeus, 1758)

970FF634-0EA7-5969-A44C-C6B0957B3792

###### Remarks.

This species was previously described by Stoliczka (1873) from Penang Hill. However, it was not found during the sampling of this study.

#### ﻿Genus *Bradybaena* H. Beck, 1837

##### ﻿ Bradybaena similaris

Taxon classificationAnimaliaGastropodaCamaenidae

(A. Férussac, 1821)

DE80B908-E07E-53F4-A8C1-CF52DABC6C89

Figs 11E , 21D

###### Material examined.

Malaysia • Penang Hill, Along Jalan Tunku Yahya Petra, Random6.; 5.4225°N, 100.2662°E; 730 m a.s.l.; 20 Jun. 2023; T.S. Liew leg.; BOR/MOL 15772.

###### Remarks.

This species was found by Stoliczka (1873) from Penang Hill. It is an introduced species and, as noted by Stoliczka (1873), it was only found in cocoa and palm plantations at the foothills and was not encountered in the forest. Similar observations were also made in this study.

#### ﻿Genus *Trichochloritis* Pilsbry, 1891

##### ﻿ Trichochloritis penangensis

Taxon classificationAnimaliaGastropodaCamaenidae

(Stoliczka, 1873)

5CFE43EA-054B-5FA1-9B9C-1ACBF373E9D0

Fig. 11F

###### Material examined.

Malaysia • Penang Hill, Air Itam, Forest near Dam, Plot.24; 5.3904°N, 100.2612°E; 380 m a.s.l.; 24 Oct. 2022; T.S. Liew, J. Dulipat leg.; BOR/MOL 15291.

###### Remarks.

This species was previously described by Stoliczka (1873) from Penang Hill. It can also be found in Perak (Maassen 2001; Foon et al. 2017).

#### ﻿Superfamily Punctoidea E. S. Morse, 1864

**Family Charopidae F. W. Hutton, 1884**

**Genus *Charopa* E. von Martens, 1860**

##### ﻿ Charopa perlata

Taxon classificationAnimaliaGastropodaCharopidae

van Benthem Jutting, 1959

C2F091FC-4E4D-5928-9942-181ED191DF08

Fig. 12A

###### Material examined.

Malaysia • Penang Hill, Moon Gate Station 5 trail, Plot.11; 5.4341°N, 100.2921°E; 110 m a.s.l.; 7 Sept. 2022; T.S. Liew, J. Dulipat, S.M. Goh leg.; BOR/MOL 15482 • Penang Hill, Teluk Bahang, Taman Rimba Teluk Bahang, Trail, Simpang 6, Plot.16; 5.4429°N, 100.2212°E; 120 m a.s.l.; 21 Oct. 2022; T.S. Liew, J. Dulipat, M.Y. Lee leg.; BOR/MOL 15560 • Penang Hill, Botanical garden, Plot.29; 5.4400°N, 100.2866°E; 75 m a.s.l.; 2 Feb. 2023; T.S. Liew, M.Y. Lee, A. Yusni leg.; BOR/MOL 15753.

**Figure 12.**
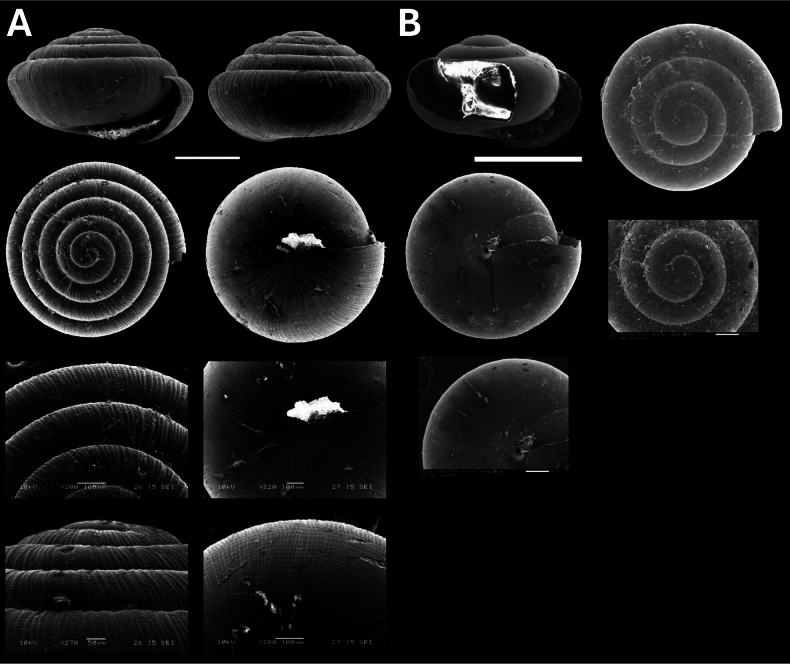
**A***Charopaperlata* van Benthem Jutting, 1959 BOR/MOL 15560 **B***Charopa* Penang sp. 2 BOR/MOL 15561. Scale bar: 0.5 mm.

###### Remarks.

This is a new record for Penang Hill. The shell characters match the description of the species originally described from Sumatra (van Benthem Jutting 1959).

##### ﻿ Charopa

Taxon classificationAnimaliaGastropodaCharopidae

Penang sp. 2

5DFD23C9-B6FA-5715-83B7-CEC81F748CBF

Fig. 12B

###### Material examined.

Malaysia • Penang Hill, The Habitat, Research trail, Plot.06; 5.4247°N, 100.2674°E; 750 m a.s.l.; 5 Sept. 2022; T.S. Liew, J. Dulipat, S.M. Goh, M.Y. Lee, M.Y. Wua leg.; BOR/MOL 15483 • Penang Hill, Teluk Bahang- Balik Pulau, Lam durian farm, Plot.13; 5.4198°N, 100.2259°E; 290 m a.s.l.; 21 Oct. 2022; T.S. Liew, J. Dulipat, M.Y. Lee leg.; BOR/MOL 15562 • Penang Hill, Teluk Bahang- Balik Pulau, Tropical fruit farm, Plot.15; 5.4160°N, 100.2200°E; 250 m a.s.l.; 21 Oct. 2022; T.S. Liew, J. Dulipat, M.Y. Lee leg.; BOR/MOL 15561 • Penang Hill, Teluk Bahang-Balik Pulau, Entrance of a durian farm, near Boulder Valley, Old rubber tree, Plot.17; 5.418°N, 100.2159°E; 180 m a.s.l.; 22 Oct. 2022; T.S. Liew, J. Dulipat leg.; BOR/MOL 15484 • Penang Hill, By Path H, Plot.33; 5.4210°N, 100.2701°E; 610 m a.s.l.; 4 Feb. 2023; T.S. Liew, A. Yusni leg.; BOR/MOL 15761.

###### Remarks.

This is a new record for Penang Hill. The shell spire is moderately raised. Teleoconch with densely spirally arranged pits above periphery, transitioning to densely placed spiral grooves on the subsequent whorls, which become more densely placed towards the periphery. Below periphery with densely placed spiral grooves that continue from the upper shell and fade away towards the umbilicus. The shell sculptures of this species are similar to *Microcystina* Penang sp. 6, but it differs by having a smaller shell size, only half the size of *Microcystina* Penang sp. 6.

#### ﻿Genus *Philalanka* Godwin-Austen, 1898

##### ﻿ Philalanka carinifera

Taxon classificationAnimaliaGastropodaCharopidae

(Stoliczka, 1873)

78F76A09-1DCE-5CED-BD00-749A2A141E8A

Fig. 13A

###### Material examined.

Malaysia • Penang Hill, The Habitat, Research trail, Plot.01; 5.4248°N, 100.2669°E; 720 m a.s.l.; 5 Sept. 2022; T.S. Liew, J. Dulipat, S.M. Goh leg.; BOR/MOL 15434 • Penang Hill, The Habitat, Habitat nature trail, Plot.05; 5.4228°N, 100.2653°E; 770 m a.s.l.; 5 Sept. 2022; T.S. Liew, J. Dulipat, S.M. Goh leg.; BOR/MOL 15435 • Penang Hill, The Habitat, Habitat nature trail, Plot.07; 5.4210°N, 100.2642°E; 760 m a.s.l.; 6 Sept. 2022; T.S. Liew, J. Dulipat, S.M. Goh, M.Y. Lee leg.; BOR/MOL 15433 • Penang Hill, The Habitat, Habitat nature trail, Plot.08; 5.4223°N, 100.2648°E; 760 m a.s.l.; 6 Sept. 2022; J. Dulipat, S.M. Goh, M.Y. Lee leg.; BOR/MOL 15437 • Penang Hill, Air Itam, Forest near Dam, Plot.24; 5.3904°N, 100.2612°E; 380 m a.s.l.; 24 Oct. 2022; T.S. Liew, J. Dulipat leg.; BOR/MOL 15436 • Penang Hill, Air Itam, Forest near Dam, Plot.25; 5.3900°N, 100.2575°E; 290 m a.s.l.; 25 Oct. 2022; T.S. Liew, J. Dulipat leg.; BOR/MOL 15440 • Penang Hill, Air Itam, Forest near Dam, Plot.26; 5.3977°N, 100.2612°E; 290 m a.s.l.; 25 Oct. 2022; T.S. Liew, J. Dulipat leg.; BOR/MOL 15438 • Penang Hill, Trail from Viaduct to Claremont, after shelter, Plot.27; 5.4205°N, 100.2724°E; 500 m a.s.l.; 26 Oct. 2022; T.S. Liew, J. Dulipat leg.; BOR/MOL 15441 • Penang Hill, Trail - Moniot Road East, Plot.28; 5.4240°N, 100.2746°E; 530 m a.s.l.; 26 Oct. 2022; T.S. Liew, J. Dulipat leg.; BOR/MOL 15439 • Penang Hill, Botanical garden, Plot.29; 5.4400°N, 100.2866°E; 75 m a.s.l.; 2 Feb. 2023; T.S. Liew, M.Y. Lee, A. Yusni leg.; BOR/MOL 15746 • Penang Hill, By Path H, Plot.33; 5.4210°N, 100.2701°E; 610 m a.s.l.; 4 Feb. 2023; T.S. Liew, A. Yusni leg.; BOR/MOL 15745 • same data as for preceding; BOR/MOL 15764.

**Figure 13.**
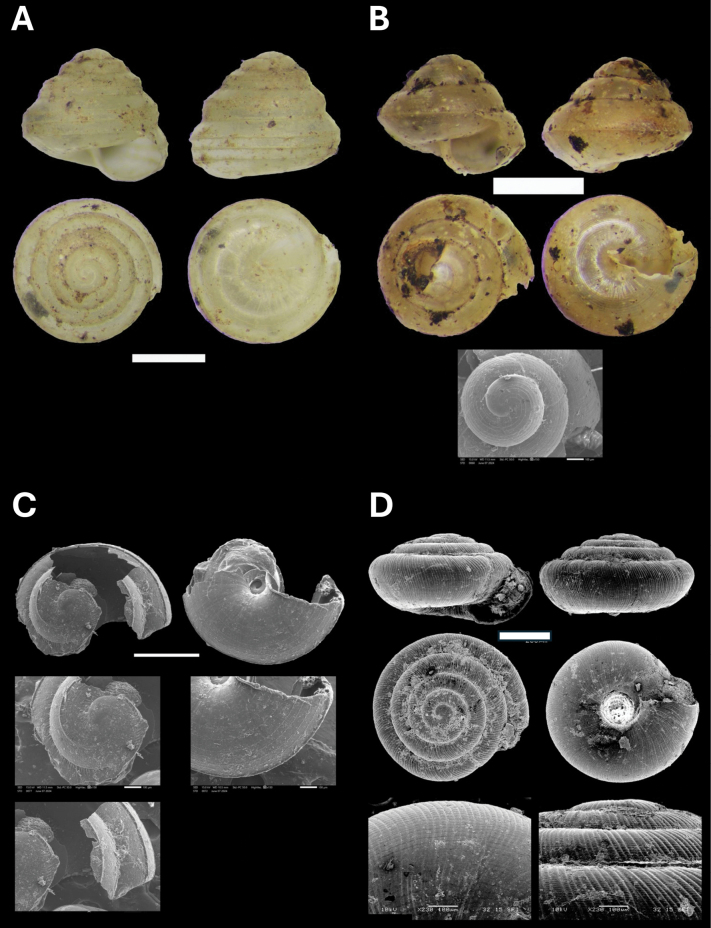
**A***Philalankacarinifera* (Stoliczka, 1873) BOR/MOL 15436 **B***Philalanka* Penang sp. 2 BOR/MOL 15539 **C***Philalankakusana* (Aldrich, 1889) BOR/MOL 15760 **D***Paralaoma* sp. BOR/MOL 15481. Scale bars: 1 mm (**A**); 0.5 mm (**B–D**).

###### Remarks.

This species was previously described by Stoliczka (1873) from Penang Hill. It differs from the other two *Philalanka* species by having three spiral ribs on the penultimate whorls.

##### ﻿ Philalanka

Taxon classificationAnimaliaGastropodaCharopidae

Penang sp. 2

5D8C50C8-E41E-5FDD-A6B0-3A760DABE37F

Fig. 13B

###### Material examined.

Malaysia • Penang Hill, Teluk Bahang, Taman Rimba Teluk Bahang, Trail, Simpang 6, Plot.16; 5.4429°N, 100.2212°E; 120 m a.s.l.; 21 Oct. 2022; T.S. Liew, J. Dulipat, M.Y. Lee leg.; BOR/MOL 15539.

###### Remarks.

This is a new record for Penang Hill. This species differs from the other two *Philalanka* species by having more than seven spiral ribs on the penultimate whorls.

##### ﻿ Philalanka kusana

Taxon classificationAnimaliaGastropodaCharopidae

(Aldrich, 1889)

B954EFA5-2453-580E-B836-3ADE22C9B077

Fig. 13C

###### Material examined.

Malaysia • Penang Hill, By Path H, Plot.33; 5.4210°N, 100.2701°E; 610 m a.s.l.; 4 Feb. 2023; T.S. Liew, A. Yusni leg.; BOR/MOL 15760.

###### Remarks.

This is a new record for Penang Hill. This species differs from the other two *Philalanka* species by having no more than two spiral ribs on the penultimate whorls.

#### ﻿Family Punctidae E. S. Morse, 1864

**Genus *Paralaoma* Iredale, 1913**

##### ﻿ Paralaoma

Taxon classificationAnimaliaGastropodaPunctidae

sp.

1702FF3E-74A0-5546-9C18-8D64A4B0E875

Fig. 13D

###### Material examined.

Malaysia • Penang Hill, Air Itam, Forest near Dam, Plot.26; 5.3977°N, 100.2612°E; 290 m a.s.l.; 25 Oct. 2022; T.S. Liew, J. Dulipat leg.; BOR/MOL 15481.

###### Remark.

This is a new record for Penang Hill.

#### ﻿Superfamily Pupilloidea W. Turton, 1831

**Family Gastrocoptidae Pilsbry, 1918**

**Genus *Gastrocopta* Wollaston, 1878**

##### ﻿ Gastrocopta palmira

Taxon classificationAnimaliaGastropodaGastrocoptidae

(Stoliczka, 1873)

4A7B8968-6654-5B02-95BF-4A3CC17C69E9

###### Remark.

Previously described and recorded by Stoliczka (1873) from Penang Hill, but not found in this study.

#### ﻿Family Valloniidae E. S. Morse, 1864

**Genus *Pupisoma* Stoliczka, 1873**

##### ﻿ Pupisoma orcella

Taxon classificationAnimaliaGastropodaValloniidae

(Stoliczka, 1873)

A2B868E4-59E8-5DD4-A190-9610E7BF6452

###### Remark.

Previous described and recorded by Stoliczka (1873) from the base of Penang Hill but not found in this study.

#### ﻿Superfamily Streptaxoidea J. E. Gray, 1860

**Family Streptaxidae J. E. Gray, 1860**

**Genus *Gulella* L. Pfeiffer, 1856**

##### ﻿ Gulella bicolor

Taxon classificationAnimaliaGastropodaStreptaxidae

(T. Hutton, 1834)

8F09C9BA-B9B8-5326-AED6-0FF22BFBDAE1

###### Remark.

Previously recorded by Stoliczka (1873) from the base of Penang Hill but not found in this study.

#### ﻿Superfamily Trochomorphoidea Mörch, 1864

**Family Chronidae Thiele, 1931**

**Genus *Kaliella* W. T. Blanford, 1863**

##### ﻿ Kaliella barrakporensis

Taxon classificationAnimaliaGastropodaChronidae

(Reeve, 1852)

C7F7EFF0-8A33-57E4-9A60-CA6C52ADFF76

Fig. 14A

###### Material examined.

Malaysia • Penang Hill, Botanical garden, Plot.29; 5.4400°N, 100.2866°E; 75 m a.s.l.; 2 Feb. 2023; T.S. Liew, M.Y. Lee, A. Yusni leg.; BOR/MOL 15739.

**Figure 14.**
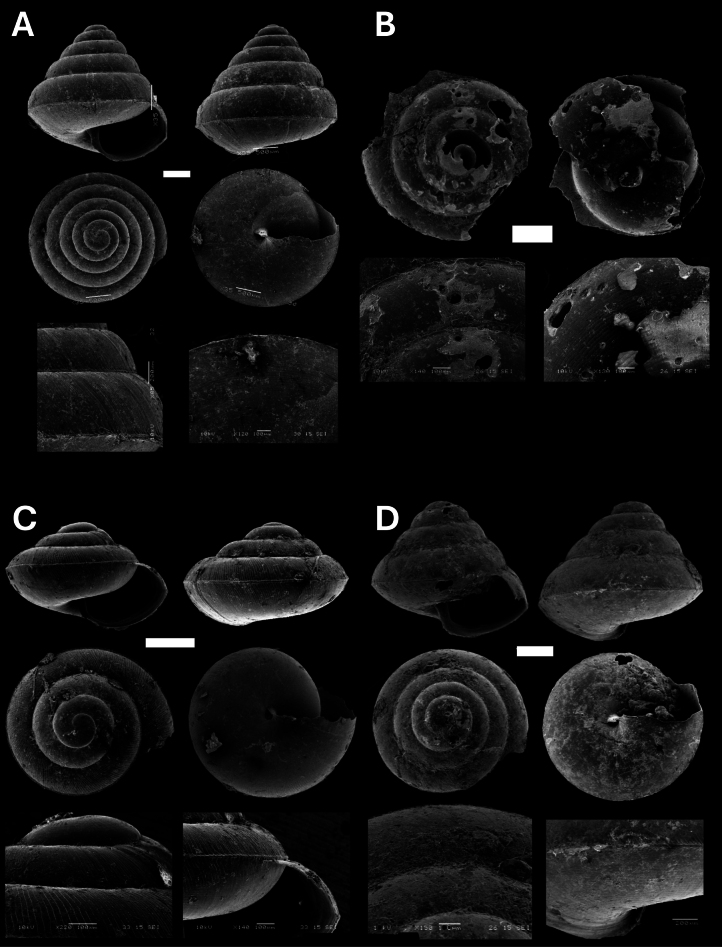
**A***Kaliellabarrakporensis* (Reeve, 1852) BOR/MOL 15739 **B***Kaliella* Penang sp. 1 BOR/MOL 15465 **C***Kaliella* Penang sp. 2 BOR/MOL 15467 **D***Kaliella* Penang sp. 4 BOR/MOL 15474. Scale bar: 0.5 mm.

###### Remarks.

This is a new record for Penang Hill. It is a widespread species in Malaysia (Vermeulen et al. 2015; Foon et al. 2017; Foon and Marzuki 2023).

##### ﻿ Kaliella

Taxon classificationAnimaliaGastropodaChronidae

Penang sp. 1

6DC1DEB9-7F25-54EE-9A70-997D70D351BC

Fig. 14B

###### Material examined.

Malaysia • Penang Hill, The Habitat, Habitat nature trail, Plot.05; 5.4228°N, 100.2653°E; 770 m a.s.l.; 5 Sept. 2022; T.S. Liew, J. Dulipat, S.M. Goh leg.; BOR/MOL 15466 • Penang Hill, The Habitat, Habitat nature trail, Plot.07; 5.4210°N, 100.2642°E; 760 m a.s.l.; 6 Sept. 2022; T.S. Liew, J. Dulipat, S.M. Goh, M.Y. Lee leg.; BOR/MOL 15465.

###### Remarks.

This is a new record for Penang Hill. The shell periphery is angular with a spiral thread visible above the suture of the penultimate whorl. Above the shell, the apical whorls are unknown, followed by regularly placed shallow radial ribs on the subsequent whorls. Below the shell, there are densely spaced spiral grooves at the periphery, which reduce towards the umbilicus.

##### ﻿ Kaliella

Taxon classificationAnimaliaGastropodaChronidae

Penang sp. 2

AE3AB794-4265-5F46-8520-EDC0774307DB

Fig. 14C

###### Material examined.

Malaysia • Penang Hill, The Habitat, Habitat nature trail, Plot.07; 5.4210°N, 100.2642°E; 760 m a.s.l.; 6 Sept. 2022; T.S. Liew, J. Dulipat, S.M. Goh, M.Y. Lee leg.; BOR/MOL 15468 • Penang Hill, The Habitat, Habitat nature trail, Plot.08; 5.4223°N, 100.2648°E; 760 m a.s.l.; 6 Sept. 2022; J. Dulipat, S.M. Goh, M.Y. Lee leg.; BOR/MOL 15469 • Penang Hill, Trail to Western hill, Km 3.6, Plot.23; 5.4203°N, 100.2517°E; 750 m a.s.l.; 24 Oct. 2022; T.S. Liew, J. Dulipat, M.Y. Lee leg.; BOR/MOL 15563 • Penang Hill, Trail - Moniot Road East, Plot.28; 5.4240°N, 100.2746°E; 530 m a.s.l.; 26 Oct. 2022; T.S. Liew, J. Dulipat leg.; BOR/MOL 15467 • Penang Hill, Moniot trail, Plot.32; 5.4165°N, 100.2574°E; 695 m a.s.l.; 4 Feb. 2023; T.S. Liew, A. Yusni leg.; BOR/MOL 15752.

###### Remarks.

This is a new record for Penang Hill. The shell is convex discus-shaped. The shell periphery is angular with a spiral thread. Above the shell, the apical whorls have shallow spiral ribs crossed by growth lines, transitioning to regularly placed prominent radial ribs on the subsequent whorls. Below the shell, radial ribs continue from above at the periphery, transitioning to spaced spiral grooves which reduce towards the umbilicus.

##### ﻿ Kaliella

Taxon classificationAnimaliaGastropodaChronidae

Penang sp. 4

27C73C89-02D0-594A-B24F-5F4699BA0D99

Fig. 14D

###### Material examined.

Malaysia • Penang Hill, The Habitat, Research trail, Plot.03; 5.4226°N, 100.2645°E; 750 m a.s.l.; 5 Sept. 2022; T.S. Liew, J. Dulipat, S.M. Goh leg.; BOR/MOL 15473 • Penang Hill, The Habitat, Research trail, Plot.06; 5.4247°N, 100.2674°E; 750 m a.s.l.; 5 Sept. 2022; T.S. Liew, J. Dulipat, S.M. Goh, M.Y. Lee, M.Y. Wua leg.; BOR/MOL 15474 • Penang Hill, The Habitat, Habitat nature trail, Plot.07; 5.4210°N, 100.2642°E; 760 m a.s.l.; 6 Sept. 2022; T.S. Liew, J. Dulipat, S.M. Goh, M.Y. Lee leg.; BOR/MOL 15470 • Penang Hill, Air Itam, Forest near Dam, Plot.25; 5.3900°N, 100.2575°E; 290 m a.s.l.; 25 Oct. 2022; T.S. Liew, J. Dulipat leg.; BOR/MOL 15471 • Penang Hill, Trail - Moniot Road East, Plot.28; 5.4240°N, 100.2746°E; 530 m a.s.l.; 26 Oct. 2022; T.S. Liew, J. Dulipat leg.; BOR/MOL 15472.

###### Remarks.

This is a new record for Penang Hill. The shell is conical in shape with an angular periphery adorned with a spiral thread. Teleoconch with elevated radial ribs above periphery that transition to densely placed fine spiral ribs on the subsequent whorls, crossed by irregular growth lines. Below periphery with less densely spaced spiral grooves at the periphery, which diminish towards the umbilicus.

##### ﻿ Kaliella

Taxon classificationAnimaliaGastropodaChronidae

Penang sp. 5

D9278023-E466-520C-8BC1-B1DBAB5AF875

Fig. 15A

###### Material examined.

Malaysia • Penang Hill, Teluk Bahang- Balik Pulau, Tropical fruit farm, Plot.15; 5.4160°N, 100.2200°E; 250 m a.s.l.; 21 Oct. 2022; T.S. Liew, J. Dulipat, M.Y. Lee leg.; BOR/MOL 15564.

**Figure 15.**
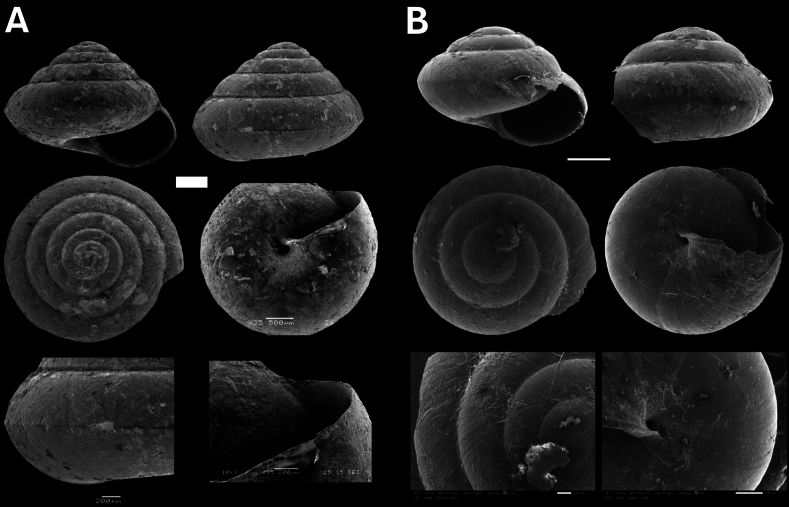
**A***Kaliella* Penang sp. 5 BOR/MOL 15564 **B***Kaliellascandens* (J. C. Cox, 1872) BOR/MOL 15476. Scale bar: 0.5 mm.

###### Remarks.

This is a new record for Penang Hill. The shell is flat conical with an angular periphery adorned with a visible spiral thread above the suture of the penultimate whorl. Teleoconch with elevated radial ribs above periphery that transition to densely placed radial ribs on the subsequent whorls. Below periphery with dense radial ribs continuing from above and transitioning to spaced spiral grooves that diminish towards the umbilicus.

##### ﻿ Kaliella scandens

Taxon classificationAnimaliaGastropodaChronidae

(J. C. Cox, 1872)

D613301E-2144-56AE-A463-2D1038E64B38

Fig. 15B

###### Material examined.

Malaysia • Penang Hill, Teluk Bahang- Balik Pulau, Tropical fruit farm, Plot.14; 5.4148°N, 100.2187°E; 260 m a.s.l.; 21 Oct. 2022; T.S. Liew, J. Dulipat, M.Y. Lee leg.; BOR/MOL 15566 • Penang Hill, Teluk Bahang- Balik Pulau, Tropical fruit farm, Plot.15; 5.4160°N, 100.2200°E; 250 m a.s.l.; 21 Oct. 2022; T.S. Liew, J. Dulipat, M.Y. Lee leg.; BOR/MOL 15565 • Penang Hill, Teluk Bahang- Balik Pulau, Lam durian farm, Plot.19; 5.4218°N, 100.2263°E; 280 m a.s.l.; 23 Oct. 2022; T.S. Liew, J. Dulipat, M.Y. Lee leg.; BOR/MOL 15476 • Penang Hill, Trail from Viaduct to Claremont, after shelter, Plot.27; 5.4205°N, 100.2724°E; 500 m a.s.l.; 26 Oct. 2022; T.S. Liew, J. Dulipat leg.; BOR/MOL 15475 • Penang Hill, Botanical garden, Plot.29; 5.4400°N, 100.2866°E; 75 m a.s.l.; 2 Feb. 2023; T.S. Liew, M.Y. Lee, A. Yusni leg.; BOR/MOL 15744 • same data as for preceding; BOR/MOL 15765.

###### Remarks.

This is a new record for Penang Hill. This species is widespread, found from Sundaland to Australia and the Pacific Islands (Vermeulen et al. 2015).

#### ﻿Family Microcystidae Thiele, 1931

**Genus *Microcystis* H. Beck, 1838**

##### ﻿ Microcystis palmicola

Taxon classificationAnimaliaGastropodaMicrocystidae

Stoliczka, 1873

4FBFF62B-9E03-5B48-88EC-1DD3E7543363

###### Remark.

This species was previously described by Stoliczka (1873) from the base of Penang Hill but was not found during the sampling of this study.

#### ﻿Genus *Vitrinopsis* C. Semper, 1873

##### ﻿ Vitrinopsis

Taxon classificationAnimaliaGastropodaMicrocystidae

sp.

BEB04B5C-0529-522A-9CBF-E2FB032FC950

Figs 16A , 21E

###### Material examined.

Malaysia • Penang Hill, The Habitat, Habitat nature trail, Plot.07; 5.4210°N, 100.2642°E; 760 m a.s.l.; 6 Sept. 2022; T.S. Liew, J. Dulipat, S.M. Goh, M.Y. Lee leg.; BOR/MOL 15120 • Penang Hill, Path B, Random1. PathB; 5.4216°N, 100.2674°E; 730 m a.s.l.; 4 Sept. 2022; T.S. Liew, J. Dulipat leg.; BOR/MOL 15108.

**Figure 16.**
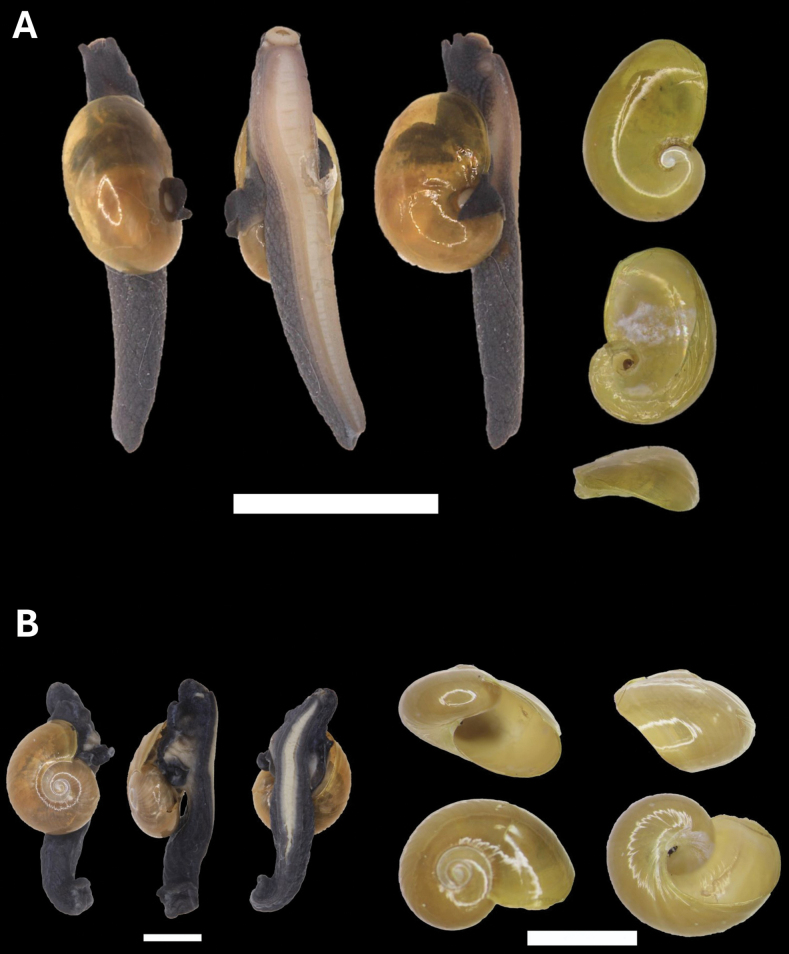
**A***Vitrinopsis* sp. BOR/MOL 15108 **B***Vitrinopsisnucleata* (Stoliczka, 1873) BOR/MOL 15238 & BOR/MOL 15233. Scale bar: 10 mm.

###### Remarks.

This is a new record for Penang Hill. This species differs from *Vitrinopsisnucleata* by having fewer than two whorls on the shell.

##### ﻿ Vitrinopsis nucleata

Taxon classificationAnimaliaGastropodaMicrocystidae

(Stoliczka, 1873)

B9D21DBE-8B5E-56A6-88F1-BA90AD0886CF

Figs 16B , 21F

###### Material examined.

Malaysia • Penang Hill, The Habitat, Habitat nature trail, Plot.04; 5.4231°N, 100.2668°E; 770 m a.s.l.; 5 Sept. 2022; T.S. Liew, J. Dulipat, S.M. Goh leg.; BOR/MOL 15115 • Penang Hill, The Habitat, Research trail, Plot.06; 5.4247°N, 100.2674°E; 750 m a.s.l.; 5 Sept. 2022; T.S. Liew, J. Dulipat, S.M. Goh, M.Y. Lee, M.Y. Wua leg.; BOR/MOL 15479 • Penang Hill, Moon Gate Station 5 trail, Plot.11; 5.4341°N, 100.2921°E; 110 m a.s.l.; 7 Sept. 2022; T.S. Liew, J. Dulipat, S.M. Goh leg.; BOR/MOL 15122 • same data as for preceding; BOR/MOL 15477 • Penang Hill, Air Itam, Forest near Dam, Plot.24; 5.3904°N, 100.2612°E; 380 m a.s.l.; 24 Oct. 2022; T.S. Liew, J. Dulipat leg.; BOR/MOL 15242 • same data as for preceding; BOR/MOL 15292 • Penang Hill, Air Itam, Forest near Dam, Plot.26; 5.3977°N, 100.2612°E; 290 m a.s.l.; 25 Oct. 2022; T.S. Liew, J. Dulipat leg.; BOR/MOL 15478 • Penang Hill, Trail from Viaduct to Claremont, after shelter, Plot.27; 5.4205°N, 100.2724°E; 500 m a.s.l.; 26 Oct. 2022; T.S. Liew, J. Dulipat leg.; BOR/MOL 15238 • Penang Hill, Trail - Moniot Road East, Plot.28; 5.4240°N, 100.2746°E; 530 m a.s.l.; 26 Oct. 2022; T.S. Liew, J. Dulipat leg.; BOR/MOL 15233 • Penang Hill, By Path H, Plot.33; 5.4210°N, 100.2701°E; 610 m a.s.l.; 4 Feb. 2023; T.S. Liew, A. Yusni leg.; BOR/MOL 15743.

###### Remark.

This species was described and recorded by Stoliczka (1873) from Penang Hill, and it can also be found in Perak (Maassen 2001; Foon et al. 2017).

#### ﻿Family Dyakiidae Gude & B. B. Woodward, 1921

**Genus *Quantula* H. B. Baker, 1941**

##### ﻿ Quantula striata

Taxon classificationAnimaliaGastropodaDyakiidae

(J. E. Gray, 1834)

EBE84A12-B781-5E71-B504-B7ECF299F136

Fig. 17A

###### Material examined.

Malaysia • Penang Hill, Botanical garden, Plot.29; 5.4400°N, 100.2866°E; 75 m a.s.l.; 2 Feb. 2023; T.S. Liew, M.Y. Lee, A. Yusni leg.; BOR/MOL 15732 • same data as for preceding; BOR/MOL 15767 • Penang Hill, Air Itam Farm, Batu Panay road, Random3. Farm; 5.3891°N, 100.2664°E; 270 m a.s.l.; 23 Oct. 2022; T.S. Liew, J. Dulipat leg.; BOR/MOL 15301.

**Figure 17.**
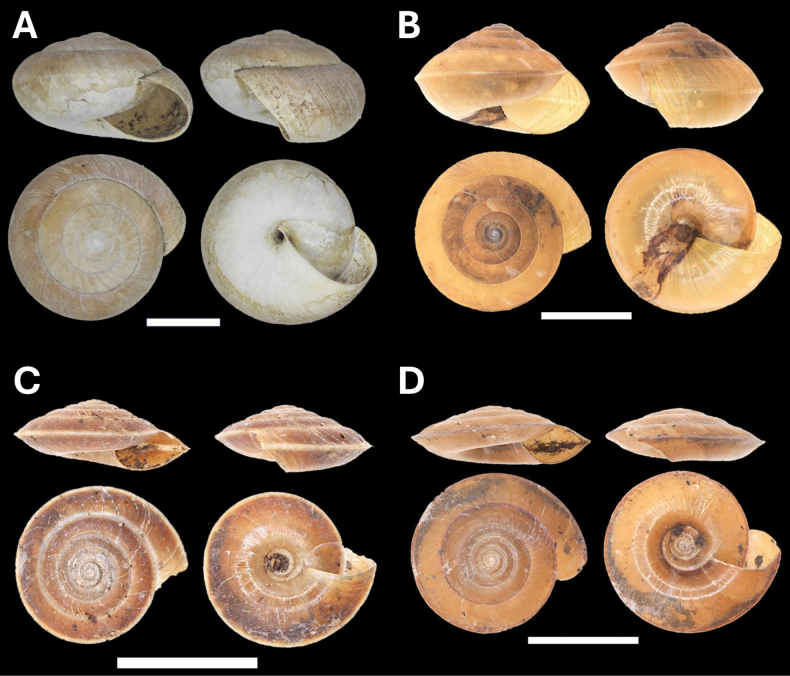
**A***Quantulastriata* (J. E. Gray, 1834) BOR/MOL 15732 **B***Pseudoplectabijuga* (Stoliczka, 1873) BOR/MOL 15734 **C***Videnacastra* (Benson, 1852) BOR/MOL 15279 **D***Videnatimorensis* (E. von Martens, 1867) BOR/MOL 15129. Scale bar: 10 mm.

###### Remarks.

One of the most common large land snails at vegetable farms of Penang Hill with a shell width of up to 28 mm. It is also known from other localities in Peninsular Malaysia, Sundaland, and the Philippines (Maassen 2001; Foon et al. 2017).

#### ﻿Genus *Pseudoplecta* Laidlaw, 1932

##### ﻿ Pseudoplecta bijuga

Taxon classificationAnimaliaGastropodaDyakiidae 

(Stoliczka, 1873)

F65149DD-AC7D-5B87-BEBE-EB29B50E64EA

Figs 17B , 22A

###### Material examined.

Malaysia • Penang Hill, The Habitat, Research trail, Plot.01; 5.4248°N, 100.2669°E; 720 m a.s.l.; 5 Sept. 2022; T.S. Liew, J. Dulipat, S.M. Goh leg.; BOR/MOL 15114 • same data as for preceding; BOR/MOL 15114 • same data as for preceding; BOR/MOL 15159 • Penang Hill, The Habitat, Research trail, Plot.02; 5.4237°N, 100.2649°E; 720 m a.s.l.; 5 Sept. 2022; T.S. Liew, J. Dulipat, S.M. Goh leg.; BOR/MOL 15113 • same data as for preceding; BOR/MOL 15197 • Penang Hill, The Habitat, Research trail, Plot.03; 5.4226°N, 100.2645°E; 750 m a.s.l.; 5 Sept. 2022; T.S. Liew, J. Dulipat, S.M. Goh leg.; BOR/MOL 15106 • Penang Hill, The Habitat, Habitat nature trail, Plot.04; 5.4231°N, 100.2668°E; 770 m a.s.l.; 5 Sept. 2022; T.S. Liew, J. Dulipat, S.M. Goh leg.; BOR/MOL 15116 • same data as for preceding; BOR/MOL 15128 • Penang Hill, The Habitat, Habitat nature trail, Plot.05; 5.4228°N, 100.2653°E; 770 m a.s.l.; 5 Sept. 2022; T.S. Liew, J. Dulipat, S.M. Goh leg.; BOR/MOL 15125 • same data as for preceding; BOR/MOL 15133 • Penang Hill, The Habitat, Research trail, Plot.06; 5.4247°N, 100.2674°E; 750 m a.s.l.; 5 Sept. 2022; T.S. Liew, J. Dulipat, S.M. Goh, M.Y. Lee, M.Y. Wua leg.; BOR/MOL 15082 • same data as for preceding; BOR/MOL 15083 • same data as for preceding; BOR/MOL 15138 • Penang Hill, The Habitat, Habitat nature trail, Plot.07; 5.4210°N, 100.2642°E; 760 m a.s.l.; 6 Sept. 2022; T.S. Liew, J. Dulipat, S.M. Goh, M.Y. Lee leg.; BOR/MOL 15086 • same data as for preceding; BOR/MOL 15089 • same data as for preceding; BOR/MOL 15146 • same data as for preceding; BOR/MOL 15431 • Penang Hill, The Habitat, Habitat nature trail, Plot.08; 5.4223°N, 100.2648°E; 760 m a.s.l.; 6 Sept. 2022; J. Dulipat, S.M. Goh, M.Y. Lee leg.; BOR/MOL 15095 • same data as for preceding; BOR/MOL 15155 • Penang Hill, Moon Gate Station 5 trail, Plot.10; 5.4305°N, 100.2913°E; 250 m a.s.l.; 7 Sept. 2022; T.S. Liew, J. Dulipat, S.M. Goh leg.; BOR/MOL 15123 • Penang Hill, Moon Gate Station 5 trail, Plot.11; 5.4341°N, 100.2921°E; 110 m a.s.l.; 7 Sept. 2022; T.S. Liew, J. Dulipat, S.M. Goh leg.; BOR/MOL 15093 • Penang Hill, Botanical garden, Plot.12; 5.4396°N, 100.2860°E; 85 m a.s.l.; 4 Sept. 2022; T.S. Liew, J. Dulipat, S.M. Goh leg.; BOR/MOL 15090 • Penang Hill, Teluk Bahang- Balik Pulau, Tropical fruit farm, Plot.15; 5.4160°N, 100.2200°E; 250 m a.s.l.; 21 Oct. 2022; T.S. Liew, J. Dulipat, M.Y. Lee leg.; BOR/MOL 15276 • Penang Hill, Teluk Bahang, Taman Rimba Teluk Bahang, Trail, Simpang 6, Plot.16; 5.4429°N, 100.2212°E; 120 m a.s.l.; 21 Oct. 2022; T.S. Liew, J. Dulipat, M.Y. Lee leg.; BOR/MOL 15268 • same data as for preceding; BOR/MOL 15274 • Penang Hill, Teluk Bahang, Taman Rimba Teluk Bahang, Trail Stesen 3, Plot.18; 5.4420°N, 100.2218°E; 150 m a.s.l.; 22 Oct. 2022; T.S. Liew, J. Dulipat leg.; BOR/MOL 15236 • same data as for preceding; BOR/MOL 15280 • Penang Hill, Teluk Bahang- Balik Pulau, Bukit Kerajaan ForestReserve next to Lam durian farm, Plot.20; 5.4199°N, 100.2265°E; 330 m a.s.l.; 23 Oct. 2022; T.S. Liew, J. Dulipat, M.Y. Lee leg.; BOR/MOL 15217 • Penang Hill, Trail to Western hill, Km 3.6, Plot.23; 5.4203°N, 100.2517°E; 750 m a.s.l.; 24 Oct. 2022; T.S. Liew, J. Dulipat, M.Y. Lee leg.; BOR/MOL 15218 • same data as for preceding; BOR/MOL 15286 • Penang Hill, Air Itam, Forest near Dam, Plot.24; 5.3904°N, 100.2612°E; 380 m a.s.l.; 24 Oct. 2022; T.S. Liew, J. Dulipat leg.; BOR/MOL 15241 • same data as for preceding; BOR/MOL 15288 • Penang Hill, Air Itam, Forest near Dam, Plot.25; 5.3900°N, 100.2575°E; 290 m a.s.l.; 25 Oct. 2022; T.S. Liew, J. Dulipat leg.; BOR/MOL 15237 • same data as for preceding; BOR/MOL 15293 • same data as for preceding; BOR/MOL 15432 • Penang Hill, Trail from Viaduct to Claremont, after shelter, Plot.27; 5.4205°N, 100.2724°E; 500 m a.s.l.; 26 Oct. 2022; T.S. Liew, J. Dulipat leg.; BOR/MOL 15234 • same data as for preceding; BOR/MOL 15297 • Penang Hill, Trail - Moniot Road East, Plot.28; 5.4240°N, 100.2746°E; 530 m a.s.l.; 26 Oct. 2022; T.S. Liew, J. Dulipat leg.; BOR/MOL 15231 • same data as for preceding; BOR/MOL 15300 • Penang Hill, Moniot trail, Plot.32; 5.4165°N, 100.2574°E; 695 m a.s.l.; 4 Feb. 2023; T.S. Liew, A. Yusni leg.; BOR/MOL 15733 • Penang Hill, By Path H, Plot.33; 5.4210°N, 100.2701°E; 610 m a.s.l.; 4 Feb. 2023; T.S. Liew, A. Yusni leg.; BOR/MOL 15734 • Penang Hill, Path B, Random1. PathB; 5.4216°N, 100.2674°E; 730 m a.s.l.; 4 Sept. 2022; T.S. Liew, J. Dulipat leg.; BOR/MOL 15150 • Penang Hill, Bukit Laksamana, Random5.; 5.4239°N, 100.2362°E; 710 m a.s.l.; 3 Feb. 2023; T.S. Liew, M.Y. Lee, A. Yusni leg.; BOR/MOL 15770.

###### Remark.

One of the most common large land snails at Penang Hill, with a shell width of up to 22 mm. It is also known from other localities in Peninsular Malaysia and Thailand (Maassen 2001; Foon et al. 2017).

#### ﻿Family Trochomorphidae Möllendorff, 1890

**Genus *Videna* H. Adams & A. Adams, 1855**

##### ﻿ Videna castra

Taxon classificationAnimaliaGastropodaTrochomorphidae

(Benson, 1852)

FE852B2D-B53A-5F8B-B63A-B1CDF1A30D2E

Fig. 17C

###### Material examined.

Malaysia • Penang Hill, Teluk Bahang-Balik Pulau, Entrance of a durian farm, near Boulder Valley, Old rubber tree, Plot.17; 5.418°N, 100.2159°E; 180 m a.s.l.; 22 Oct. 2022; T.S. Liew, J. Dulipat leg.; BOR/MOL 15279.

###### Remarks.

This species was also previously recorded by Stoliczka (1873). It is similar to *Videnacantoriana* but has more regularly expanded whorls. This species is also recorded from mainland Peninsular Malaysia, India, and Thailand (Maassen 2001; Foon et al. 2017).

##### ﻿ Videna cantoriana

Taxon classificationAnimaliaGastropodaTrochomorphidae

(W. H. Benson, 1861)

8AC0A91B-8E8C-51B9-86F3-62F7A68756D6

###### Remarks.

This species was previously recorded and described from Penang Hill (at ca 2000 feet a.s.l.) by Stoliczka (1873). It was not found in this study.

##### ﻿ Videna timorensis

Taxon classificationAnimaliaGastropodaTrochomorphidae

(E. von Martens, 1867)

314D032F-DF35-5160-973C-50248E09BC9D

Fig. 17D

###### Material examined.

Malaysia • Penang Hill, The Habitat, Habitat nature trail, Plot.04; 5.4231°N, 100.2668°E; 770 m a.s.l.; 5 Sept. 2022; T.S. Liew, J. Dulipat, S.M. Goh leg.; BOR/MOL 15129.

###### Remarks.

This species was also previously recorded by Stoliczka (1873). It differs from the other two *Videna* species of Penang Hill by having a wider, more open umbilicus, approximately one-third of the shell width.

#### ﻿Superfamily Veronicelloidea J. E. Gray, 1840

**Family Rathouisiidae Heude, 1885**

**Genus *Atopos* Simroth, 1891**

##### ﻿ Atopos tourannensis

Taxon classificationAnimaliaGastropodaRathouisiidae

(Souleyet, 1852)

DA698A05-17A6-58D9-8728-1C376E2F7279

Figs 18A , 22B

###### Material examined.

Malaysia • Penang Hill, Teluk Bahang-Balik Pulau, Entrance of a durian farm, near Boulder Valley, Old rubber tree, Plot.17; 5.418°N, 100.2159°E; 180 m a.s.l.; 22 Oct. 2022; T.S. Liew, J. Dulipat leg.; BOR/MOL 15227.

**Figure 18.**
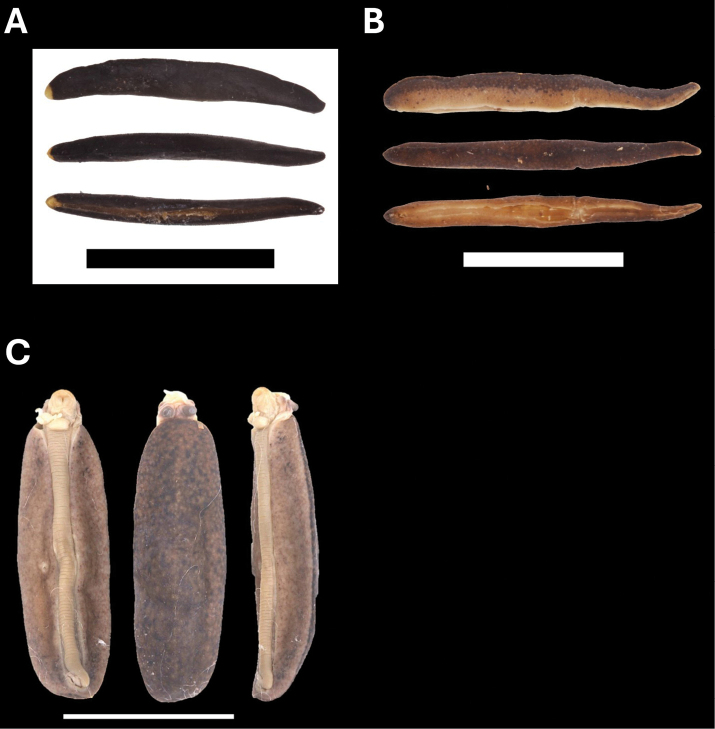
**A***Atopostourannensis* (Souleyet, 1852) BOR/MOL 15227 **B***Atopospunctata* Collinge, 1902 BOR/MOL 15228 **C***Laevicaulisalte* (A. Férussac, 1822) BOR/MOL 15773. Scale bar: 10 mm.

###### Remarks.

This is a new record for Penang Hill. This species differs from *Atopospunctata* by having a black mantle with white areas at both ends (head and tail) of the mantle.

##### ﻿ Atopos punctata

Taxon classificationAnimaliaGastropodaRathouisiidae

Collinge, 1902

26F32015-AC1F-5D07-907D-21E35021BEF4

Figs 18B , 22C

###### Material examined.

Malaysia • Penang Hill, Trail from Viaduct to Claremont, after shelter, Plot.27; 5.4205°N, 100.2724°E; 500 m a.s.l.; 26 Oct. 2022; T.S. Liew, J. Dulipat leg.; BOR/MOL 15228.

**Figure 19.**
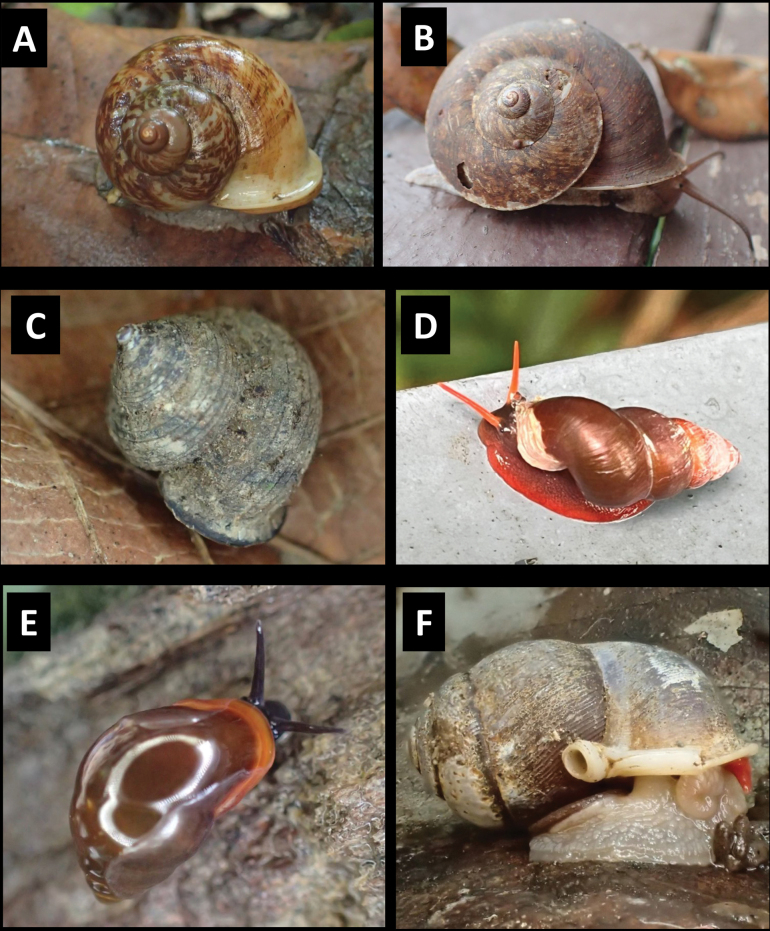
**A***Cyclophorusmalayanus* (W. H. Benson, 1852) **B***Cyclophorusperdixborneensis* (Metcalfe, 1854) **C***Lagocheilustrochoides* (Stoliczka, 1872) **D***Coptocheilussectilabris* (A. Gould, 1844), photo credit Mei Yi Lee **E***Pupinaaureola* Stoliczka, 1872 **F***Rhaphauluslorraini* L. Pfeiffer, 1856.

###### Remarks.

This species was also previously recorded by Stoliczka (1873). It differs from *Atopostourannensis* by having a brown mantle.

**Figure 20.**
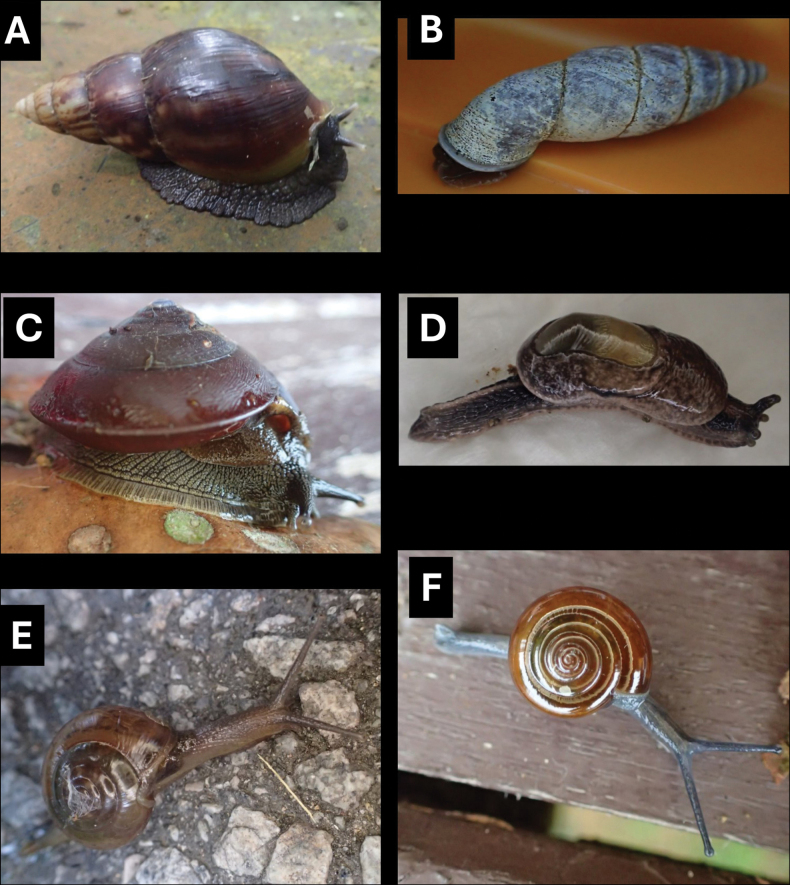
**A***Lissachatinafulica* (Bowdich, 1822) **B***Oospirapenangensis* (Stoliczka, 1873) **C***Hemiplectacymatium* (L. Pfeiffer, 1856) **D***Parmarionmartensi* Simroth, 1893 **E***Tanychlamysindica* (Godwin Austen, 1883) **F***Tanychlamys* Penang sp. 1.

#### ﻿Family Veronicellidae J. E. Gray, 1840

**Genus *Laevicaulis* Simroth, 1913**

##### ﻿ Laevicaulis alte

Taxon classificationAnimaliaGastropodaVeronicellidae

(A. Férussac, 1822)

2366391C-699B-585F-880E-2F12A77C6FE5

Figs 18C , 22D

###### Material examined.

Malaysia • Penang Hill, Path B, Random1. PathB; 5.4216°N, 100.2674°E; 730 m a.s.l.; 4 Sept. 2022; T.S. Liew, J. Dulipat leg.; BOR/MOL 15111 • Penang Hill, Plaza, Random2. Plaza; 5.4247°N, 100.2689°E; 740 m a.s.l.; 6 Sept. 2022; T.S. Liew, J. Dulipat leg.; BOR/MOL 15112 • Penang Hill, Along Jalan Tunku Yahya Petra, Random6.; 5.4225°N, 100.2662°E; 730 m a.s.l.; 20 Jun. 2023; T.S. Liew leg.; BOR/MOL 15773.

**Figure 21.**
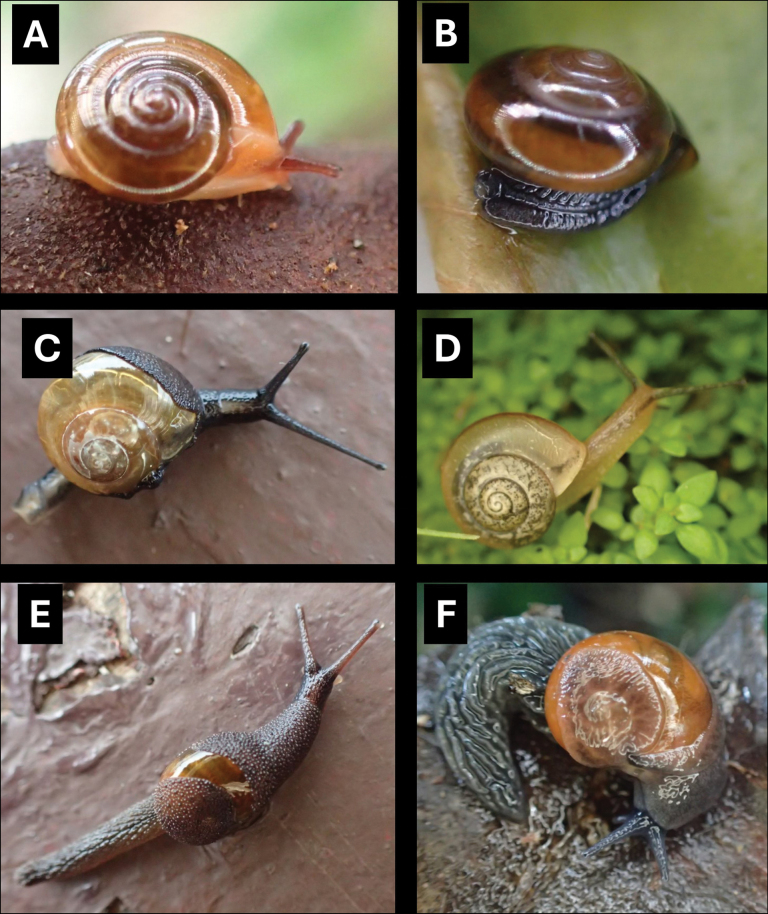
**A***Tanychlamys* Penang sp. 2 **B***Tanychlamystersa* (Issel, 1874) **C***Helicarionpermolle* Stoliczka, 1873 **D***Bradybaenasimilaris* (A. Férussac, 1821) **E***Vitrinopsis* sp. **F***Vitrinopsisnucleata* (Stoliczka, 1873).

###### Remarks.

This is a new record for Penang Hill. So far, this species has only been found in human-altered habitats. The most reliable method for determining species identity involves detailed anatomical examination. However, as this specimen is a juvenile, some identifying features may not be fully developed. Based on the available evidence, we tentatively identify it as *L.alte*.

**Figure 22.**
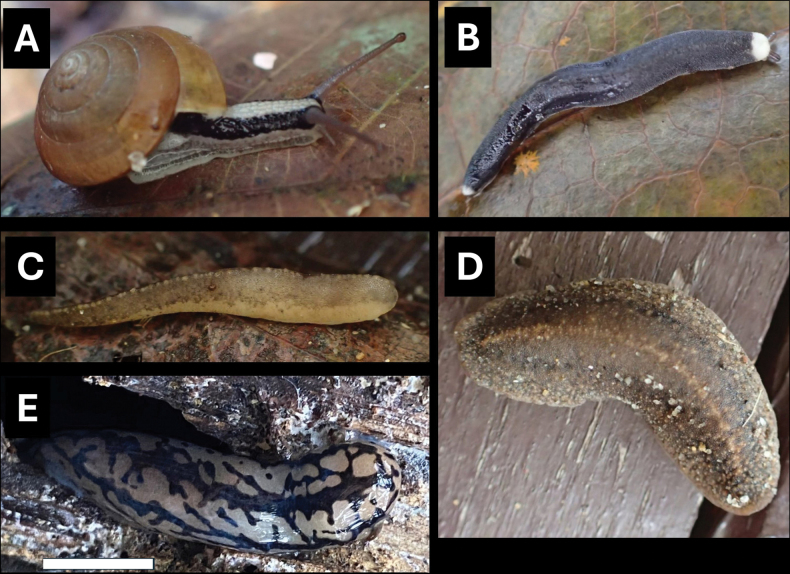
**A***Pseudoplectabijuga* (Stoliczka, 1873) **B***Atopostourannensis* (Souleyet, 1852) **C***Atopospunctata* Collinge, 1902 **D***Laevicaulisalte* (A. Férussac, 1822) **E***Meghimatiumpictum* (Stoliczka, 1873), photo credit Xin Wei Ooi.

#### ﻿Genus *Semperula* Grimpe & Hoffmann, 1924

##### ﻿ Semperula birmanica

Taxon classificationAnimaliaGastropodaVeronicellidae

(Theobald, 1864)

D14BDCBA-5E4A-5825-BFE2-E56B02D887C6

###### Remark.

Old record by Stoliczka (1873).

## ﻿Discussion

### ﻿Sampling completeness and inventory of land snails

Our work has led to a significant increase of the number of recorded species from Penang Hill since Stoliczka (1872, 1873): 34 new records of 66 species recorded. Many of the new records are micro-snails from genera such as *Kaliella*, *Microcystina*, *Diplommatina*, *Charopa*, *Ditropopsis*, *Paralaoma*, and *Philalanka*. This discovery can be attributed to our greatly improved collecting techniques. Others are singletons, and the fact that they have not been collected before may be due to the patchiness of their distribution.

The species richness estimates by iNEXT suggested that the species richness of Penang Hill is 94 species. This indicates that, while our systematic sampling design is effective in capturing a substantial proportion of the land snail diversity in the area, considerably more sampling effort will be needed to obtain a complete overview of the land snail community at Penang Hill. The number of species recorded in Penang Hill increased from 32 to 66, demonstrating a high species richness compared to that typically found in limestone habitats (e.g., Foon et al. 2017; Foon and Marzuki 2023) or in very high mountains for non-limestone areas (e.g., 82 species on Mount Kinabalu (Malaysia) at 4095 m a.s.l. and 66 species on Mount Tambuyukon (Malaysia) at 2579 m a.s.l. in Liew et al. 2010). By comparing the historical records with those species found in this study, seven of the newly recorded species are found to be alien species: these are not micro-snails which could have been missed by Stoliczka (1872, 1873). Almost all the species were found in the disturbed forests and orchards, and on a few occasions recorded along the trails in undisturbed forest. This observation aligns with the findings by Cowie (1998), who noted that non-indigenous land snails and slugs are generally associated with disturbed, low- and mid-elevation habitats. This could suggest the possibility that these species were introduced after the sampling made by Stoliczka in 1869. Another possibly introduced species is *Macrochlamys* Penang sp. 1, which is relatively large, and currently one of the most common snail species (found in 17 of the 33 sampled plots) that, if it would have been present in Stoliczka’s time, would not have been overlooked by him. Unlike the confirmed alien species that are affiliated with disturbed habitats, this species can be found in disturbed and undisturbed habitats, which raises concerns about their potential impact on the native ecosystem.

### ﻿Species diversity and composition among plots of different habitat types

The habitat types (undisturbed forest, disturbed forest, and orchards) do exhibit differences in species richness, though it is not statistically significant. Orchards and undisturbed forest have a similar number of species, but with different species composition, revealed by the cluster analysis and NMDS. Disturbed habitats seem to have higher numbers of species and could be due to the mid-disturbance effect on species richness (Čiliak et al. 2024). The intermediate levels of disturbance, such as man-made structures and modified vegetation with ornamental plants, in the disturbed forest plots seemed to create favourable conditions for non-native species while the remaining original vegetation and forest can support the native species: both conditions contribute to greater diversity of land snail species.

While there were no clear patterns of grouping based on elevation or habitat types, the plots in orchards exhibited some similarity in terms of species composition. We found five species have strong associations to disturbed forest areas and orchards, while most of the other species do not show clear preferences for a certain habitat. This observation suggests that human-modified landscapes, such as orchards, might create specific microhabitats that support a particular group of land snail species.

## ﻿Conclusions

Overall, this study significantly increases our knowledge of land snail diversity in non-limestone areas of Peninsular Malaysia. Additionally, the material collected in this study are valuable for future taxonomic research in this region, given that Penang Hill is the type locality for 19 species newly described by Stoliczka (1872, 1873). Penang Hill remains one of the most important historical sites for biodiversity research and continues to harbour a rich biodiversity.

## Supplementary Material

XML Treatment for Alycaeus gibbosulus

XML Treatment for Cyclophorus malayanus

XML Treatment for Cyclophorus perdix perdix

XML Treatment for Cyclotus solutus

XML Treatment for Ditropopsis

XML Treatment for Lagocheilus

XML Treatment for Lagocheilus trochoides

XML Treatment for Lagocheilus striolatus

XML Treatment for Opisthoporus penangensis

XML Treatment for Diplommatina

XML Treatment for Diplommatina crosseana

XML Treatment for Coptocheilus sectilabris

XML Treatment for Pupina aureola

XML Treatment for Rhaphaulus lorraini

XML Treatment for Allopeas clavulinum

XML Treatment for Allopeas gracile

XML Treatment for Lissachatina fulica

XML Treatment for Paropeas tchehelense

XML Treatment for Subulina octona

XML Treatment for Meghimatium pictum

XML Treatment for Oospira penangensis

XML Treatment for Phaedusa filicostata

XML Treatment for Hemiplecta cymatium

XML Treatment for Microcystina

XML Treatment for Microcystina

XML Treatment for Microcystina

XML Treatment for Microcystina

XML Treatment for Microcystina

XML Treatment for Parmarion martensi

XML Treatment for Tanychlamys indica

XML Treatment for Tanychlamys

XML Treatment for Tanychlamys

XML Treatment for Tanychlamys stephoides

XML Treatment for Tanychlamys tersa

XML Treatment for Helicarion permolle

XML Treatment for Amphidromus atricallosus

XML Treatment for Amphidromus perversus

XML Treatment for Bradybaena similaris

XML Treatment for Trichochloritis penangensis

XML Treatment for Charopa perlata

XML Treatment for Charopa

XML Treatment for Philalanka carinifera

XML Treatment for Philalanka

XML Treatment for Philalanka kusana

XML Treatment for Paralaoma

XML Treatment for Gastrocopta palmira

XML Treatment for Pupisoma orcella

XML Treatment for Gulella bicolor

XML Treatment for Kaliella barrakporensis

XML Treatment for Kaliella

XML Treatment for Kaliella

XML Treatment for Kaliella

XML Treatment for Kaliella

XML Treatment for Kaliella scandens

XML Treatment for Microcystis palmicola

XML Treatment for Vitrinopsis

XML Treatment for Vitrinopsis nucleata

XML Treatment for Quantula striata

XML Treatment for Pseudoplecta bijuga

XML Treatment for Videna castra

XML Treatment for Videna cantoriana

XML Treatment for Videna timorensis

XML Treatment for Atopos tourannensis

XML Treatment for Atopos punctata

XML Treatment for Laevicaulis alte

XML Treatment for Semperula birmanica

## ﻿Additional information

### Author ORCIDs

Mei-Yi Lee https://orcid.org/0009-0005-8200-3508

Thor-Seng Liew https://orcid.org/0000-0002-9437-5924

### Data availability

All of the data that support the findings of this study are available in the main text or Supplementary Information.

## Supplementary materials

Supplementary material 1 Species plot data matrix for the 33 standard sampling sites at Penang Hill (plot locations, habitat types, and elevation details in Table 1) Data type csv https://binary.pensoft.net/file/1270458This dataset is made available under the Open Database License (http://opendatacommons.org/licenses/odbl/1.0/). The Open Database License (ODbL) is a license agreement intended to allow users to freely share, modify, and use this Dataset while maintaining this same freedom for others, provided that the original source and author(s) are credited.Author: Thor-Seng Liew
